# A versatile information retrieval framework for evaluating profile strength and similarity

**DOI:** 10.1101/2024.04.01.587631

**Published:** 2024-04-02

**Authors:** Alexandr A. Kalinin, John Arevalo, Loan Vulliard, Erik Serrano, Hillary Tsang, Michael Bornholdt, Bartek Rajwa, Anne E. Carpenter, Gregory P. Way, Shantanu Singh

**Affiliations:** 1 Imaging Platform, Broad Institute of MIT and Harvard, Cambridge MA, USA; 2 Systems Immunology and Single-Cell Biology, German Cancer Research Center (DKFZ), Heidelberg, Germany; 3 Department of Biomedical Informatics, University of Colorado School of Medicine, Aurora CO, USA; 4 Bindley Bioscience Center, Purdue University, West Lafayette IN, USA

## Abstract

In profiling assays, thousands of biological properties are measured in a single test, yielding biological discoveries by capturing the state of a cell population, often at the single-cell level. However, for profiling datasets, it has been challenging to evaluate the phenotypic activity of a sample and the phenotypic consistency among samples, due to profiles’ high dimensionality, heterogeneous nature, and non-linear properties. Existing methods leave researchers uncertain where to draw boundaries between meaningful biological response and technical noise. Here, we developed a statistical framework that uses the well-established mean average precision (mAP) as a single, data-driven metric to bridge this gap. We validated the mAP framework against established metrics through simulations and real-world data applications, revealing its ability to capture subtle and meaningful biological differences in cell state. Specifically, we used mAP to assess both phenotypic activity for a given perturbation (or a sample) as well as consistency within groups of perturbations (or samples) across diverse high-dimensional datasets. We evaluated the framework on different profile types (image, protein, and mRNA profiles), perturbation types (CRISPR gene editing, gene overexpression, and small molecules), and profile resolutions (single-cell and bulk). Our open-source software allows this framework to be applied to identify interesting biological phenomena and promising therapeutics from large-scale profiling data.

## Introduction

Today, the study of complex diseases and biological processes at the systems level increasingly relies on the use of multiplex and high-throughput experiments. One particular experimental design, known as “profiling,” has emerged as a powerful approach to characterize biological functions, classify patient subpopulations, and identify promising therapeutic targets ^1–3^. A typical profiling experiment measures hundreds to tens of thousands of features of a biological system simultaneously. The measurements can report on bulk properties or offer single-cell resolution, depending on the experimental design and research question asked. Thus, they convey information about the molecular (e.g., genomic, epigenomic, transcriptomic, proteomic, or metabolomic) or cellular (e.g., morphological, spatial, viability across cell lines) status of the system ^4–6^. Some profiling experiments can be executed at high-throughput scale, where biological samples are subjected to various perturbations, usually chemical compounds or genetic reagents ^6–11^. Ultimately, by combining high-dimensional readouts, diverse biological samples, and multiple perturbations, profiling can reveal the mechanisms of biological processes and potential therapeutic avenues.

By casting a wide and systematic net, profiling experiments are often the first step in prioritizing interesting biology to study in more detail. However, there are challenges introduced by the high dimensionality and heterogeneous nature of profiling datasets, impacting the ability to distinguish perturbations associated with interesting biology from less relevant phenomena, including technical variation. Typically, priority is given to perturbations with higher replicability and activity, i.e., those that both occur consistently across technical replicates and exhibit phenotypes significantly different from a negative control or a baseline. However, evaluating these properties in large, complex data that contain multiple profiles for both controls and perturbations remains a major challenge. Likewise, it is also difficult to distinguish between samples that genuinely resemble each other (such as chemicals with the same mechanism or genes with the same function) as compared to those that only appear similar due to being prepared in the same plate or batch.

The most commonly used methods for evaluating profiles are either based on univariate metrics or, more recently, machine learning (ML)^12^. In general, univariate metrics are calculated on a per-feature basis, overlooking the joint effect of features on the outcomes. Even the more recent multidimensional extensions ^13,14^ still rely on assumptions that each feature’s measurements are normally distributed and that, for the most part, observed phenomena are linear and not co-dependent ^15,16^. Thus, these approaches broadly oversimplify the behavior of biological systems^17^. On the other hand, ML strategies use a classifier to sort measured phenotypes into distinct groups, where biological replicability and activity are determined by a better ability to classify samples from controls or each other. However, these methods are not readily adopted by the community for this purpose, because in addition to the high computational cost of creating numerous pairwise classifiers, ML strategies face the dual challenges of limited replicates in biological studies and overfitting, which requires extensive model evaluation, analytical design (e.g. model selection, train/test splitting), and parameter tuning. Additionally, ML approaches can overfit confounding variables, such as batch effects, which can be hard to ascertain, especially if the model is not fully explainable^18^. This precludes the selection of a single set of parameters that applies to all scenarios, because these choices are (and should be) influenced by each particular experimental design or application. Evaluation that does not require extensive parameter tuning and adapts easily across various experimental designs, such as those relying on distance metrics alone, is therefore preferred.

To overcome these issues, we developed a statistical framework and open-source software for comprehensive assessment of profile strength and similarity. It is based on the well-established mean average precision (mAP) as a single data-driven evaluation metric that we adapt to many useful scenarios in profiling. mAP assesses the probability that samples of interest will rank highly on a list of samples rank ordered by some distance or similarity metric. We demonstrate how mAP can determine whether perturbations or groups of perturbations differ from controls and/or from each other. We provide a detailed description of mAP properties in this context and a method for assigning statistical significance to mAP scores such that resulting *p*-values can be used to filter profiles by phenotypic activity and/or consistency. We show the advantages of mAP over an existing metric using simulated data and illustrate the utility of mAP on a variety of real-world datasets, including image-based (Cell Painting^19^), protein (nELISA^6^), and mRNA (Perturb-seq^7–10^) profiling data, some at the single-cell level, and involving several perturbation types. We provide a Python package for efficient calculation of mAP scores and corresponding *p*-values that enables easy and scalable application of our method to other datasets. We expect that the mAP framework we provide will streamline hypothesis generation and improve hit prioritization from a wide range of large-scale, high-throughout biological profiling data.

## Results

### General AP calculation framework

**Figure 1.**
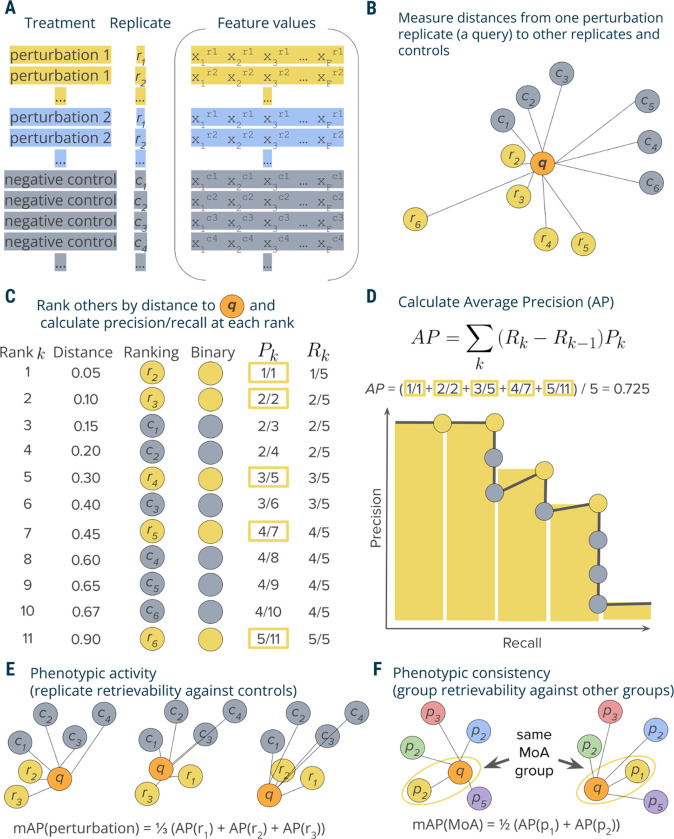
Schematic overview of mAP calculation workflow. **A:** A typical output of a profiling experiment contains multiple replicate profiles for each perturbation and controls. **B**: To measure average precision per perturbation replicate, we selected one replicate profile as a query and measured distances to its other replicates and controls. **C**: Profiles were then ranked by decreasing similarity (increasing distance) to the query; the rank list was converted to binary form and used to calculate precision *P*_*k*_ and recall *R*_*k*_ at each rank *k*. **D**: Average precision was calculated by averaging precision values over those ranks *k* containing perturbation replicates, which corresponds to a non-interpolated approximation of the area under the precision-recall curve. **E**: By applying this procedure to each perturbation replicate, we calculated a set of AP scores that were then averaged to obtain a mAP score for a perturbation’s *phenotypic activity*. **F**: One can also apply the same framework to retrieving groups of perturbations with the same biological annotations (rather than groups of replicates of the same perturbation)—for example, compounds that share the same mechanism of action (MoA)—by calculating the mAP score per each group of perturbations (MoA).

Mean average precision (mAP) is a performance metric routinely used in information retrieval and machine learning, particularly in the context of ranking tasks ^20,21^. mAP measures the ability of a given query to retrieve other samples from the same group (“correct” samples) from a collection that also includes samples from another group (“incorrect” samples). In this work, we used mAP to indicate the degree to which profiles from one group exhibit greater intra-group similarity compared to their similarity with the profiles from a second group. We evaluated its behavior to compare any two groups of high-dimensional profiles, such as replicates of the same perturbation against controls, perturbations annotated with related phenotypic activities against other perturbations, one experimental batch of samples against another, etc. We showed how mAP can be calculated for diverse applications, contingent on the specific definition of profile groupings and given a similarity ranking of profiles relative to the query (based on a distance function of choice). It is important to note that mAP computation requires a minimum of two profiles in each group. In this section, we present the framework for calculating mAP (Figure 1) and outline various tasks where it can be effectively used.

In a typical profiling experiment, both perturbations and controls are represented by multiple biological replicates, i.e., the same perturbation is replicated across multiple wells and, depending on the experiment scale, even multiple plates and batches (Figure 1A). These replicate profiles can be obtained directly at the well level or first measured at the single-cell level and then aggregated. To illustrate the mAP calculation process, this discussion will focus on quantifying the *phenotypic activity* of a single perturbation based on its replicate retrievability, i.e., retrieving its replicates against a control group.

Profiles can be viewed as points in a high-dimensional feature or representation space, where the closeness between pairs corresponds to profile similarity. Profile similarity can be assessed by measuring the distance between these points defined by any relevant distance function, such as cosine, Euclidean, Mahalanobis, etc., such that a larger distance between points indicates lower similarity and vice versa. The proposed mAP framework is agnostic to the choice of this distance metric. Here, we used cosine distance due to its ability to identify related samples from biological perturbational data based on the similarity of their change patterns, rather than the extent of these changes ^22–24^. Following a typical information retrieval workflow^21^, we began by designating one profile from a replicate group as a query and measuring distances between the query and the rest of this perturbation’s replicates as well as control replicates (Figure 1B).

We rank profiles by their decreasing similarity to the query, such that the most similar profile is at the top of the list (Figure 1C). We then convert this ranked list to a binary form by replacing perturbation replicates with ones (these are “correct matches”, i.e., expected to be more similar to the query) and controls with zeros (they are ”incorrect matches”, i.e., expected to be less similar to the query). In an ideal scenario where a perturbation produces a strong signal that is technically replicable, all perturbation replicates are more similar to each other than to controls and, hence, will appear on the top of the list. However, in practice, it is often challenging to detect differences from controls, especially given the presence of technical variation (Supplementary Figure S1). Having a binary rank list allows calculating precision and recall at each rank *k*. Precision at rank *k* is the fraction of ranks 1 to *k* that contain correct matches (ones). Recall at rank *k* is the fraction of the correct matches (ones) across ranks from 1 to *k*.

Although there are multiple possible ways to aggregate precision and/or recall values, we chose calculating average precision (AP) because of its statistical properties. It has an underlying theoretical basis as it corresponds to the area under the precision-recall curve^20^, it allows a probabilistic interpretation^25^, it has a natural top-heavy bias^26^ (top-ranked correct matches contribute more than low-ranked), it is highly informative for predicting other metrics such as R-precision^27^, and finally, it results in good performance when used as an objective in learning-to-rank methods^28^. Although many formulations of AP exist^29^, we calculate the conventional non-interpolated AP score as the average value of precision over those ranks *k* that contain correct matches^20^ (Figure 1D).

### mAP calculation and statistical significance

By applying the aforementioned procedure to compute the Average Precision (AP) for each replicate of a perturbation, we acquire a collection of AP scores (Figure 1E). Averaging these scores yields the perturbation-level mean Average Precision (mAP) score^20^. This score effectively quantifies the phenotypic activity of a perturbation, reflecting the average extent to which its replicate profiles were more similar to each other compared to control profiles (Figure 1E).

Our mAP framework is general and can thus be applied to many settings, as described in subsequent sections. In addition to measuring phenotypic activity for each perturbation as just described, we also used it to assess the *phenotypic consistency* of multiple group members annotated with common biological mechanisms or modes of action (Figure 1F). In this setting, we first aggregated replicate profiles at the perturbation level (for example, by taking the median value for each feature of single cells). We then applied the mAP framework to quantify to what extent perturbations with related biological annotations produce profiles that resemble each other compared to other perturbations in the experiment. By using other perturbations for the null distribution instead of negative controls, we assessed the biological specificity of each group of profiles relative to other samples. We also adapted mAP to assess technical batch and plate effects, as explained in the section on applications to real data.

In all cases, we determined the statistical significance of the mAP score using permutation testing, a common method for significance determination when distribution of the test statistic is unknown (for example, not known to follow a normal distribution). This approach is frequently applied in biological data analysis, including high-throughput screening^14^. Under the null hypothesis, we assume that both perturbation and control replicates were drawn from the same distribution. To generate mAP distribution under the null hypothesis, we repeatedly reshuffle the rank list and recalculate mAP. The *p*-value is then calculated according to standard practices for permutation-based methods, defined as the fraction of permutation-derived mAP values that are greater than or equal to the original mAP value. This approach aligns with the interpretation of significance values in parametric statistical analyses, where a nominal significance cutoff of 0.05 is typically used. Finally, these *p*-values are corrected for multiple comparisons using False Discovery Rate (FDR) control methods, such as Benjamini–Hochberg procedure^30^ or its alternatives^31^. We refer to the percentage of calculated mAP scores with a corrected *p*-value below 0.05 as the percent retrieved (see Methods*:* Assigning significance to mAP scores *for details)*.

We therefore concluded that the mAP framework, as described and applied, could assess various qualities of high-dimensional profiling data by quantifying the similarity within a group of profiles in contrast to their similarity to another group. Unlike existing solutions, mAP is completely data-driven, does not involve complex calculations or parameter tuning, and is independent of the underlying nature of the observations or distance measures. It is flexible across various experimental designs and offers a robust means to ascertain the statistical significance of the observed similarities or differences.

### mAP outperforms mp-value on simulated data

We next sought to rigorously assess and compare the performance of our mAP framework against existing metrics using simulated data, where profile characteristics could be carefully controlled. Among established approaches, we selected the multidimensional perturbation value^14^ (mp-value) for comparison because it is multivariate by design, can be applied to any two groups of profiles, and has been shown to outperform other approaches, including univariate and clustering-based, in a simulation study with a similar design^14^. It is based on a combination of principal component analysis, Mahalanobis distance, and permutation testing to determine whether two groups of profiles differ from each other. By comparing our framework with this established method in controlled scenarios that mimic real-world experimental conditions, we aimed to evaluate mAP’s potential as a more effective tool for analyzing subtle differences in profiling data.

We conducted simulations to evaluate mAP performance by generating perturbation and control profiles such that each perturbation had 2 to 4 replicates, in experiments with 12, 24, or 36 control replicates (in all combinations). The simulated profiles varied in feature size, ranging from 100 to 5,000, representing a typical range for various kinds of profiles, particularly after feature reduction or selection. We generated control profile features using a standard normal distribution (μ = 0, σ = 1). For perturbation replicates, a certain proportion of features (ranging from 1% to 64%) were sampled from a normal distribution with a shifted mean (μ = 1, σ = 1), while the rest were drawn from the standard normal distribution. All simulated profiles were rescaled to unit norm such that for each profile, the sum of all squared feature values equals to 1. Following the previously described method, we calculated mAP scores and corresponding *p-*values at the perturbation level to assess phenotypic activity for each perturbation (see Figure 1E). We measured performance by calculating recall as the proportion of the 100 simulated perturbations for which each metric achieved statistical significance (p < 0.05).

**Figure 2.**
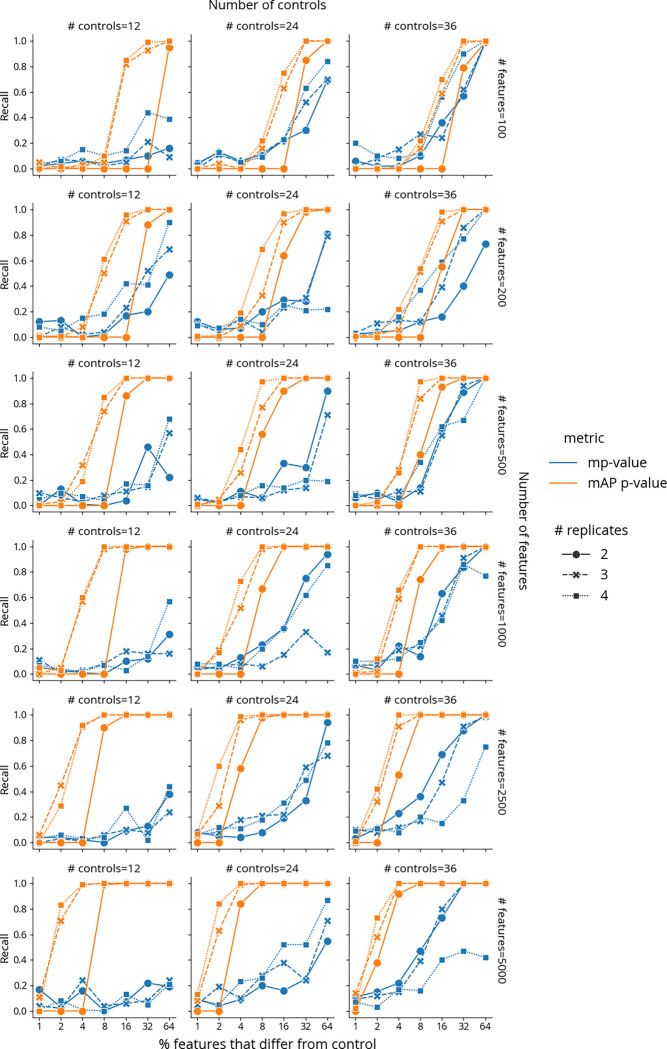
Benchmarking performance of mAP *p*-value (orange) and mp-value (blue) for phenotypic activity on simulated data. Recall indicates the percentage of 100 simulated perturbations under each condition that were called accurately by each method (as distinguishable from negative controls, or not). The horizontal axis probes what proportion of the features in the profile was different from controls. Marker and line styles indicate different numbers of replicates per perturbation (# replicates of 2, 3, and 4). Columns correspond to the different number of controls (# controls of 12, 24, and 36). Rows correspond to different profile sizes (# features being 100, 200, 500, 1000, 2500, and 5000). Note the binary exponential scaling on the x axis.

mAP consistently outperformed or matched mp-value in accurately distinguishing between perturbation profiles and controls in most simulated scenarios, demonstrating its capability to discern subtle differences (Figure 2). Our findings highlighted that both mAP and mp-value were sensitive to the experimental design, including the number of replicates and controls, the dimensionality (number of features) of the dataset, and the proportion of features that were perturbed. As expected, a decrease in the number of replicates and controls generally led to reduced performance for both metrics. mAP’s recall rate consistently improved with an increase in the number of features and the proportion of perturbed features. This trend highlights mAP’s adaptability to high-dimensional data, a critical advantage in handling the vast feature spaces typical in modern profiling assays. In contrast to mAP, the performance of mp-value was less stable and often demonstrated a decline in recall with an increase in the number of features.

The only instance where mAP performance was clearly worse than mp-value occurred when each perturbation had only 2 replicates. This limitation, however, stems not from the mAP calculation framework itself but from the constraints of the permutation testing approach when applied to a rank-based statistic in such scenarios. Specifically, with only 13 elements in the binary rank list (one perturbation replicate and 12 controls), achieving a *p*-value < 0.05 is unlikely due to the insufficient number of possible rearrangements.

Taken together, our findings reveal mAP’s superior performance in most scenarios, highlighting its potential as a more effective and adaptable tool for biological data analysis compared to existing sophisticated methods like mp-value. Specifically, we found mAP could sensitively detect subtle differences between samples, in the context most relevant to large high-dimensional profiling datasets: scenarios when the number of features was much larger than the number of replicate profiles per sample. Unlike mp-value, mAP does not require complex matrix operations to achieve that sensitivity.

### mAP captures diverse properties of real-world morphological profiling data with both genetic and chemical perturbations

Next, we demonstrated the versatility of the mAP framework through its application to different tasks on real-world data, evaluating the effects of selected preprocessing methods and experimental designs. We began with image-based profiles of genetic perturbations and tested several ways mAP can be used for tasks beyond ranking perturbations by their phenotypic activity. We chose our published “Cell Health” dataset of Cell Painting images of CRISPR-Cas9 knockout perturbations of 59 genes, targeted by 119 guides in three different cell lines^32^. We used a subset of 100 guides that had exactly six replicates (two replicates in three different plates) in each cell line.

**Figure 3.**
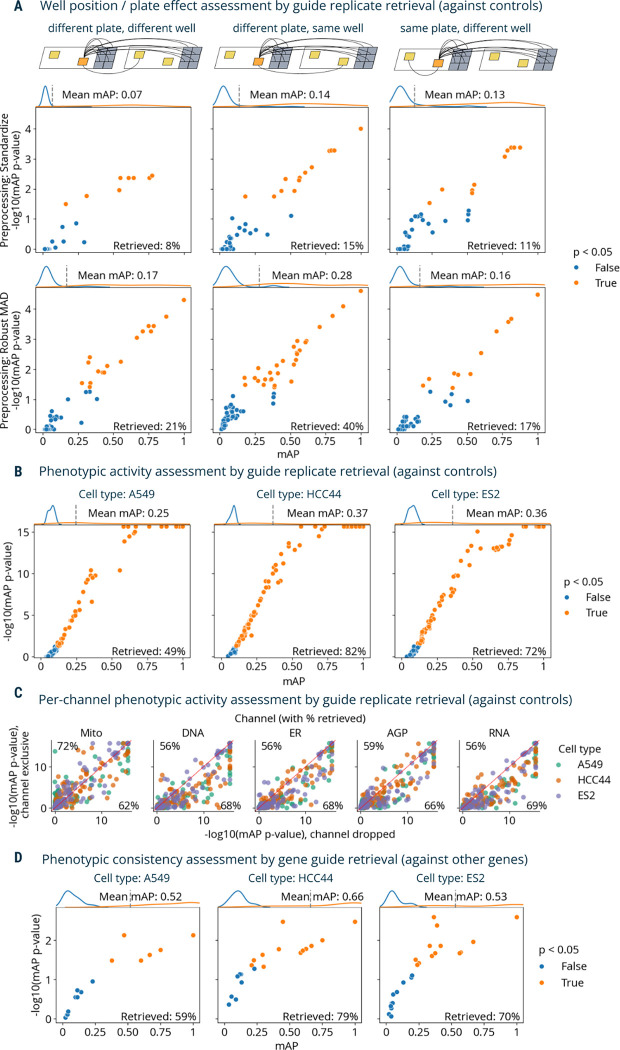
mAP applied to morphological profiling of CRISPR-Cas9 knockout perturbations (Cell Health dataset). **A**: mAP is calculated to assess well position and batch (individual plate) effects by comparing guide phenotypic activity against controls in three scenarios (replicates of a guide across different plates and well positions; replicates of a guide across different plates, but in the same well position; and replicates of a guide within the same plate, but in different well position) and two data preprocessing methods (standardization and robust MAD). Results are shown for a subset of an arbitrary pair of plates as a proof-of-concept. **B**: mAP is calculated to assess the phenotypic activity of perturbations by guide replicate retrievability against controls in three cell lines individually (68% retrieved on average across all three cell lines). Results included all three replicate plates available per cell line. **C**: mAP *p*-values calculated to assess influence of individual fluorescent channels on guide phenotypic activity against controls by either dropping a channel or including only that single channel (percent retrieved is shown for each axis), these results can be compared to 68% retrieved when all channels’ data is available (on average across all three cell lines, as in **B**). **D**: mAP is calculated to assess the phenotypic consistency of guides annotated with related target genes (against guides annotated with other genes) in three cell lines individually.

We assessed how phenotypic activity (estimated via replicate retrievability mAP against non-targeting cutting controls^33^) (Figure 1E) can be used to evaluate three aspects of data quality: well position effects, batch effects, and phenotypic activity of each guide (Figure 3A–C). These tasks only differ based on how replicates are grouped (i.e., which sample wells count as a correct or incorrect match).

First, we calculated mAP to assess well-position and batch effects (considering each plate as a separate batch) across two different data preprocessing methods (Figure 3A) in A549 cells. The first preprocessing method included data standardization by subtracting means from feature values and dividing them by variance using the whole dataset. Alternatively, we used a robust version of standardization, which replaces mean and variance with median and median absolute deviation, correspondingly, and is applied on a per-plate basis (“Robust MAD”). We leveraged the fact that each guide had replicates in different well positions and plates to formulate three profile groupings for well position and batch effect assessment. The first group only included profiles derived from different plates and well position; the second group only included profiles from the same well position, but different plates; and the third group only included profiles from the same plate, but different wells. In each scenario, we retrieved grouped profiles against negative controls from both plates.

In the absence of well-position effects and batch effects, these three tasks should demonstrate similar phenotypic activity mAP. However, as expected, the data reveals both well-position and batch effects (Figure 3A): with standardized profiles, retrieval of replicates in a different well position and different plate performed the worst (8% retrieved), while sharing the same plate or well position showed better results (11% and 15% retrieved, correspondingly). By contrast, using per-plate Robust MAD as a preprocessing method improved retrieval of profiles from a different well position and different plate (21% retrieved) to a larger extent than it did for the same plate, different well test (17% retrieved). But it also inflated retrieval of profiles that share the same well position in different plates (40% retrieved), demonstrating that well position effects were not addressed by this pre-processing and may affect downstream analyses. We used Robust MAD for preprocessing for all subsequent analysis given its better performance on a challenging task (retrieving from different well position, different plate). This example showcased the flexibility of the mAP framework for assessing technical variation in data and evaluating methods to correct for it by grouping profiles according to experimental properties.

Next, we assessed each CRISPR guide’s phenotypic activity by replicate retrievability (Figure 1E) in three cell types. In this case, all six replicates per guide were retrieved against non-targeting controls. Mean mAP scores and percent retrieved varied 0.16–0.28 and 49–82% by cell line, respectively (Figure 3B), showing that mAP captures cell context-dependent differences of each guide’s phenotypic activity, though potentially confounded by well-position and batch effects. We observed similar results using an alternate negative control, wells that were not perturbed at all (Supplementary Figure S2A). Significance of mAP was somewhat negatively correlated with CERES scores^34^ (Supplementary Figure S2B), a measure of gene essentiality derived from viability experiments, confirming that many perturbations that impact viability also impact morphology^35^, though one would expect many exceptions, for example, for genes that are not expressed well in the given cell type.

Then, we evaluated the mAP framework for characterizing contributions of different fluorescent channels by calculating metrics for each single channel individually (Figure 3C, Y axis); the mitochondria channel proved the most independently useful for retrieving guide replicates against controls. In most cases, dropping a channel (Figure 3C, X axis) only slightly diminished retrieval performance, a useful guide for researchers wanting to swap out a channel for a particular marker of interest. In a similar fashion, we assessed the contributions of different feature types extracted from different cell compartments and found (Supplementary Figure S2C) that, for example, excluding AreaShape features dropped the percentage of retrieved guides below 50%. Removing Granularity or Texture features resulted in ~70% and ~80% guides retrieved, correspondingly, while using Granularity features alone had a higher percentage than using just Texture (except for Cell compartment), thus, Granularity appears to be more informative than Texture in all three cell types, which can hint at what phenotypic responses were distinguishing for this gene set as a whole.

We next assessed phenotypic consistency of CRISPR guides that targeted the same gene by retrieving them against guides that targeted other genes (similar to Figure 1F), to see whether guides targeting the same gene yielded a consistent and relatively distinctive phenotype. First, we aggregated each guide’s six replicates by taking the median value for each feature. Then, we filtered guides that did not pass the significance threshold for phenotypic activity in each cell type (Figure 3B) to remove profiles that could not be confidently distinguished from controls. There were two aggregated guide profiles per gene annotation, which we retrieved against guide profiles of other genes (2 “replicates” vs 118 “controls” using the terms of Figure 2). As a result, mAP values ranged 0.52–0.66 with 59–70% of genes retrieved (Figure 3D).

Finally, we applied the mAP framework to other perturbation types (small molecules and gene overexpression, rather than CRISPR-Cas9 knockouts), to assess their phenotypic activity (Supplementary Figure S3). We used the dataset “cpg0004”^36^, which contains Cell Painting images of 1,327 small-molecule perturbations of A549 human cells and the JUMP Consortium’s “cpg0016[orf]” dataset^37^ of U2OS cells treated with 15,136 overexpression reagents (open reading frame - ORFs), encompassing 12,602 unique genes, including controls, making it the largest dataset in this study in terms of number of perturbations. In both cases, we first calculated mAP to assess the phenotypic activity of each perturbation by replicate retrievability against controls, which resulted in 34% of small molecules retrieved for cpg0004 (Supplementary Figure S3A) and 39% of ORFs retrieved (Supplementary Figure S3B). Subsequently, we filtered out perturbations that did not pass the phenotypic activity threshold and aggregated the rest on a per-perturbation basis by computing the median value for each feature across replicates and then. Finally, we calculated mAP to assess phenotypic consistency (a measure of whether profiles capture true biological meaning, captured here by public annotations). We tested for phenotypic consistency among small molecules that were annotated as targeting the same gene (cpg0004) or among ORFs encoding genes that produce proteins that were annotated as interacting with each other, per the mammalian CORUM database^38^ cpg0016[orf]. For many reasons, including imperfect and incomplete annotations, annotations from differing cell contexts, and the non-comprehensive nature of any particular assay (even a profiling assay), we expected success rates to be low. For cpg0004, 35% of small molecules’ target genes showed consistent phenotypic similarity (Supplementary Figure S3C), while for cpg0016[orf] it was 21% of assessed protein complexes (Supplementary Figure S3D).

These results demonstrated that the proposed mAP framework can be used for assessing various properties of real-world morphological profiling data created with both genetic and chemical perturbations. By changing how profile groupings are defined, mAP can be used for multiple purposes: to characterize technical variation in data, to evaluate methods to address them, to determine the contributions of specific fluorescent channels or measured feature types, and to ultimately select and rank perturbations by their phenotypic activity and consistency for potential downstream analyses.

### mAP quantifies strength and similarity of protein and single-cell mRNA profiling data

To demonstrate the applicability of the mAP framework beyond image-based profiling, we applied it to other modalities, including transcriptomics and proteomics.

The first dataset contained proteomic profiles from a 191-plex nELISA, a high-throughput, high-plex assay designed for quantitative profiling of the secretome^6^, which was performed in A549 cells across 306 well-characterized compound perturbations from the Broad Institute’s drug repurposing library^39^. This dataset also had matching Cell Painting morphological profiles imaged from the same physical samples whose supernatants were nELISA-profiled.

**Figure 4.**
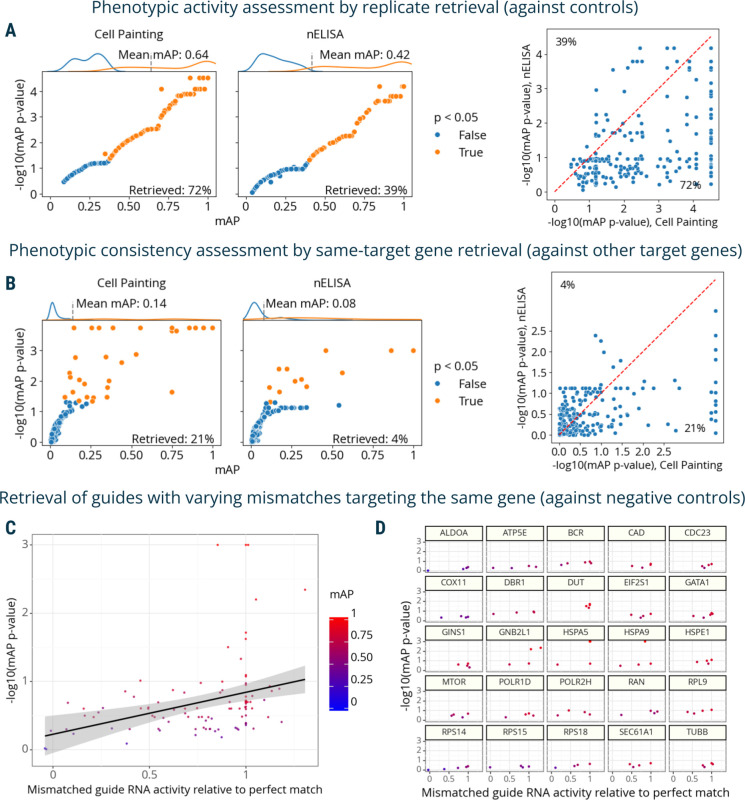
mAP applied to proteomic and mRNA profiling. **A**: mAP is calculated to assess the phenotypic activity of compounds by replicate retrievability against controls in matching Cell Painting and nELISA profiling data. **B**: mAP is calculated to assess the phenotypic consistency by retrieving phenotypically active compounds annotated with the same gene target in matching Cell Painting and nELISA profiling data (note: the nELISA panel includes 191 targets including cytokines, chemokines, and growth factors which are not expected to respond well in these convenience samples from a prior study, because there is no immune stimulation and the A549 cells used have limited secretory capacity). **C:** mAP is calculated to assess the mRNA profile-based phenotypic activity of a mismatched CRISPRi guide from a Perturb-seq experiment (y-axis) and correlate it with the guide’s activity relative to a perfectly matching guide for that gene (x-axis). **D**: A subset of the data from panel **C** is presented, with several genes highlighted individually to demonstrate the variation from gene to gene.

First, we used mAP to assess phenotypic activity via replicate retrievability for both assays. This analysis resulted in 72% of compounds being retrieved using Cell Painting and 39% with nELISA (Figure 4A); the smaller percentage is likely due to A549 cells’ limited secretory capacities, the absence of immune stimulation, and a mismatch between pathways targeted by small molecules and nELISA readouts^6^. Among the compounds showing significant activity, we further calculated mAP to assess phenotypic consistency by identifying compounds annotated with the same target gene. This analysis yielded 22% retrieval for Cell Painting and 4% for nELISA (Figure 4B), again likely due to the limitations in the original experimental design that was not ideal for secretome profiling. This comparison validated mAP’s utility in comparing two different profiling assays, offering valuable insights for planning future studies, for example, selecting an appropriate cell type for a particular assay.

Finally, we used mAP to evaluate a Perturb-seq^7–10^ mRNA profiling dataset of single cells treated with CRISPRi. The experiment assessed how single-guide RNAs (sgRNAs) containing mismatches to their target sites attenuate expression levels of target genes^40^. Specifically, 25 genes involved in a diverse range of essential cell biological processes were targeted with 5–6 mismatched sgRNAs, covering the range from full to low activity, and 10 nontargeting controls. Each mismatched guide was characterized by its activity levels relative to the perfectly matched sgRNA targeting the same gene^40^. We compared mAP scores to sgRNA relative activity, expecting that guide mismatches that disrupt activity levels to a larger extent should have mRNA profiles that are less easily distinguishable from controls. We indeed observed an overall correlation between mAP scores for a sgRNA’s mRNA profile similarity and its relative activity levels, (Figure 4C), with more nuanced differences in correlations for specific genes (Figure 4D).

These applications demonstrate mAP’s robustness in quantifying the strength and similarity of image, protein, and mRNA profiles, affirming its broad utility across diverse profiling assays.

### mAP captures subtle phenotypic impacts of perturbations at the single-cell resolution

Single-cell profiling has become increasingly feasible, providing detailed and nuanced insights into the complex nature of biological systems, which are often obscured in bulk analyses. To illustrate the mAP framework’s utility in analyzing single-cell data, we applied it to two distinct single-cell profiling datasets.

**Figure 5.**
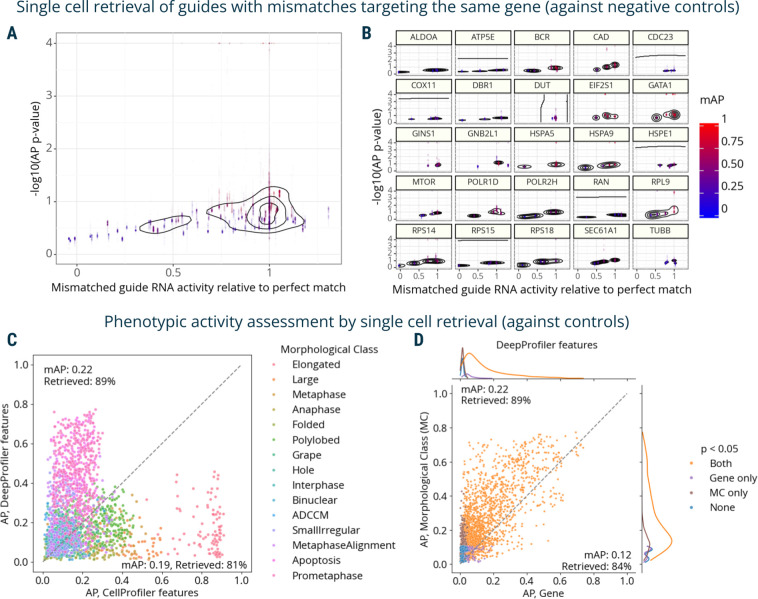
mAP applied to single-cell mRNA and imaging data. **A**: AP scores are calculated to assess the single-cell mRNA profile-based phenotypic activity of a mismatched CRISPRi guide from a Perturb-seq experiment (y-axis) and correlate it with the guide’s activity relative to a perfectly matching guide for that gene (x-axis). **B**: A subset of the data from panel **A** is presented, with several genes highlighted individually to demonstrate the variation from gene to gene.. **C**: AP scores are calculated to evaluate the power of CellProfiler and DeepProfiler features to classify multiple phenotypic classes in Mitocheck morphological data. AP scores capture the ability to retrieve single cells annotated with the same morphological class against negative controls. **D:** Mitocheck data, correlation between mAP scores for retrieving single cells annotated with the same morphological class versus gene, for DeepProfiler features. MC: morphological class.

First, we repeated the analysis of the Perturb-seq mRNA profiling dataset^40^ (Figure 4C–D) on the single-cell level. The relationship between single-cell AP scores and relative activity levels closely recapitulated those observed in the bulk profiles (Figure 5A), while per-gene visualization revealed varied levels of heterogeneity across individual cells (Figure 5B).

The second dataset called “Mitocheck” contained cell images of genome-wide gene silencing by RNA interference^41^. We used a subset of these images, in which almost 3,000 cells were manually annotated with observed morphological classes and processed by either CellProfiler^42^ or DeepProfiler^43^ feature extractors to create single-cell morphological profiles. After filtering out cells that failed quality control or were out of focus, the subset contained 2456 single-cell profiles annotated with 15 morphological classes across 60 genes.

We used replicate retrievability against non-targeting controls to compare phenotypic activity of single-cell CellProfiler- and DeepProfiler-derived profiles grouped by morphological classes and target genes. Both feature extraction methods showed on average similar performance, retrieving morphological annotations with 0.19–0.22 mAP and 81–89% retrieved (Figure 5C, Supplementary Figure S4A). Interestingly, however, their performance varied across individual classes (Figure 5C, Supplementary Figure S4B), indicating complementarity in phenotypes that are characterized more informatively with one approach than the other. Although performance dropped for both methods in the target gene retrieval task (Supplementary Figure S4C), the decrease was more severe for CellProfiler features (0.07 mAP, 64% retrieved) compared to DeepProfiler (0.12 mAP, 84% retrieved). When comparing performance across both tasks, CellProfiler features overall demonstrated more variability across the range of mAP scores (Supplementary Figure S4D) compared to more consistent results of DeepProfiler (Figure 5D).

This analysis underscores the ability of the mAP framework to discern phenotypic variability and heterogeneity inherent in single-cell data, revealing both the strengths and complementary nature of different feature extraction methodologies.

## Discussion

High-throughput profiling experiments have shown great promise in elucidating biological functions, patient subpopulations, and therapeutic targets. However, the high dimensionality and heterogeneity of profiling datasets present a significant obstacle for traditional methods in evaluating data quality and identifying meaningful relationships among profiles. Our work advances this domain by proposing a comprehensive statistical and computational framework using mean average precision (mAP) to assess profile strength and similarity. The mAP framework identifies phenotypically active perturbations by the replicability of repeated experiments as compared to negative controls. It also measures phenotypic consistency among different perturbations that share biological similarity, such as chemical mechanisms of action and gene-gene relationships, by retrieving perturbation groups. The framework can be applied to image-based, protein, and gene expression profiling datasets created with either genetic and chemical perturbations. mAP assessments can shed light on the presence of technical variation in data (e.g., plate layout and batch effects), suitability of experimental design (cell type or fluorescent channel selection), and data processing methods (feature extraction). This adaptability makes mAP a valuable tool for comparing different profiling methods and enhancing the interpretation of high-throughput experiments.

At its core, the mAP framework is based on calculating a well-established evaluation metric on the rank list of nearest neighbors to a given profile. Unlike most existing alternatives^12^, this procedure is robust to outliers, fully agnostic to the nature of data, and does not make distributional, linearity, or sample size assumptions. With its top-heavy bias, average precision is similar to other recently proposed metrics that emphasize early discovery in ranking assessment for drug discovery^44–46^, but those metrics require careful parameter tuning that can be tricky. Unlike the AP^25^, those metrics cannot be interpreted in terms of probability even if they are bounded by [0, 1]^45^. If only k top ranked perturbations are of interest, requiring the rank list to be thresholded (for example, when the goal is to see how often the correct profile would be in the top k results), AP can be easily replaced by Precision@*k*.

Still, the mAP framework has limitations. As a rank-based metric, it is robust to deviations from typical assumptions for parametric methods, but it cannot reflect differences in effect size beyond perfect separability between two groups that are being compared. To overcome this limitation, future studies could explore extending mAP to accommodate graded rank lists^26^, moving beyond binary classifications. Further, the effectiveness of the mAP framework, like other methods based on nearest neighbors, is contingent upon choosing an appropriate measure of profile dissimilarity (a distance metric). A less-informative distance metric would impair mAP’s performance, but this is a trade-off for the framework’s flexibility, as mAP can be utilized with any appropriate dissimilarity measure to suit different analyses and data types. Finally, the permutation testing approach used for significance assessment has limitations when dealing with datasets that have a small number of replicates or controls. This is an inherent statistical constraint that highlights the importance of having adequate experimental replicates and controls for robust statistical analysis.

In conclusion, the mAP framework presents a powerful strategy for evaluating data quality and biological relationships among samples in high-throughput profiling. It adjusts to various data types and perturbations, and is robust to the complexities of real-world biological data. It can be effectively used to improve methods and prioritize perturbations for further studies with the potential to streamline the discovery of mechanisms and therapeutic targets in complex biological systems.

## Methods

### mAP calculation

In general, the mAP framework can be used to compare any two groups of high-dimensional profiles by retrieving profiles from one group (“query group”) against another group (“reference group”).

Given a group of N reference profiles and a group of M query profiles, we calculate non-interpolated AP^20^ for each query profile as following:

1. out of M query profiles, select one profile i;
2. measure distances from the query profile i to all other (M-1)+N profiles in both groups;
3. rank-order (M-1)+N profiles by increasing distance to the query profile i (decreasing similarity);
4. for each rank k going top-down the list, if k contains another query profile (true positive we term a “correct match”, i.e. not a reference), calculate *precision@k* for this rank k;
5. when done, average calculated precisions to obtain the AP value.

More formally, average precision for profile i is calculated as:

$AP_{i}=\frac{1}{(M-1)+N}\sum_{k=1}^{(M-1)+N}\;g_{k}P_{k}$, where

$g_{k}$ equals 1 if rank k contains a correct match (True Positive) and 0 if otherwise,

$P_{k}=\frac{TP_{k}}{k}$ is precision at rank k (precision@k),

$TP_{k}$ is the number of all query profiles (all Positives) retrieved up to rank k.

More conveniently, AP can be expressed via relative change in recall:

$AP_{i}=\sum_{k=1}^{(M-1)+N}\;\left(R_{k-1}-R_{k}\right)P_{k}$, where $P_{k}$ is the same as above and

$R_{k}=\frac{TP_{k}}{M-1}$ is recall at rank k (recall @k), $R_{0}=0$, which replaces both $g_{k}$ and dividing by M-1.

Then, mean AP (mAP) for the whole query group can be calculated through aggregation of individual query profile APs by taking a mean:

$mAP=\frac{1}{M}\sum_{i=1}^{M}\;AP_{i}$, where M is the number of profiles in the query group.

### Assigning significance to mAP scores

We estimate the statistical significance of a mAP score with respect to a random baseline using a permutation testing approach, a non-parametric, assumption-free method for testing the null hypothesis of sample exchangeability. Under the null hypothesis, we assume that profiles in both query and reference groups were drawn from the same distribution. To generate mAP distribution under the null hypothesis, we repeatedly reshuffle the rank list and recalculate mAP. The *p*-value is then calculated as the fraction of the null that is greater than or equal to the mAP score. This approach aligns with the interpretation of significance values in parametric statistical analyses, where a nominal significance cutoff of 0.05 is typically used. When we compare mAP scores of multiple query groups, we correct corresponding *p*-values for multiple comparisons using the Benjamini–Hochberg procedure^30^. We refer to the percentage of calculated mAP scores with a corrected *p*-value below 0.05 as the percent retrieved.

### mAP for phenotypic activity and consistency assessment

We applied the mAP framework to assess phenotypic activity and consistency.

We assess phenotypic activity of a single perturbation by calculating mAP for replicate retrievability, i.e. the ability to retrieve a group of perturbation’s replicates (query group) against a group of control profiles (reference group). At this stage, a replicate profile typically means an aggregation of single-cell profiles (e.g., across all cells in a single well). By imposing additional conditions, we defined various groups of replicates for a given perturbation. For example, we used phenotypic activity to evaluate the presence of plate effects by comparing mAP score for retrieving replicates from the same plate vs from different plates. After calculating mAP scores for all perturbations, they can be compared and ranked in terms of their phenotypic activity.

We also use mAP to assess the phenotypic consistency of multiple perturbations annotated with common biological mechanisms or modes of action (query group) against perturbations with different annotations (reference). When computing phenotypic consistency, each perturbation’s replicate profiles are first aggregated into a consensus profile by taking the median of each feature to reduce profile noise and improve computational efficiency.

Let’s consider a dataset containing perturbations annotated with mechanisms of action. For example, a group of P compounds is annotated with $MoA_{1}$, and the rest Q compounds are annotated with various other MoA labels.

Then the mAP for the ${MoA}_{1}$ group of P perturbations can be computed as following:

1. select one perturbation profile from this group, e.g. $P_{i}$;
2. measure distances from $P_{i}$ to all other (P-1)+Q profiles in both groups;
3. rank-order (P-1)+Q profiles by decreasing similarity w.r.t to $P_{i}$
4. going top-down the list, if the rank k contains a perturbation profile from the same group P, calculate *precision@k* for this rank k
5. when done, average calculated precisions by summing them up and dividing by P-1
6. repeat the process for all i=1…P and average obtained APs to calculate mAP_*P*_

The resulting value mAP_*P*_ will indicate how internally consistent (has high mAP for retrieving perturbations from itself) this group of perturbations annotated with ${MoA}_{1}$ is compared to other perturbations. This example can be easily extended to an arbitrary number of perturbation groups (e.g., compound MoAs). The same process can also be repeated using each set of perturbations as a query group. This will result in obtaining mAP scores for all groups of perturbations in the dataset and can be used to rank them by phenotypic consistency or estimate the consistency of the whole dataset by aggregating them (e.g., by averaging).

### Extension to multiple labels

When considering groups of perturbations, a single perturbation can belong to multiple groups simultaneously. For example, a compound can have multiple annotations, such as genes whose products are targeted by the compound, or mechanisms of action of this compound. Then AP can be calculated by considering a single annotation group at a time. In the example below, we assume having per-perturbation aggregated consensus profiles.

Let’s consider a dataset containing consensus profiles of P perturbations, with each perturbation annotated with “labels” from 1…T, where T is the number of all possible labels in the dataset.

Then for a label and one of the perturbations annotated with it, AP can be calculated as:

1. select one label t from T possible options
2. select one perturbation profile $p_{t}$ out of $P_{t}$ *perturbations annotated with this label* (query)
3. rank-order the rest of profiles (P-1)+1 by similarity w.r.t to $p_{t}$
4. going top-down the list, if the rank k contains a perturbation profile that is also annotated with the label t, calculate *precision@k* for this rank k
5. when done, average calculated precisions by summing them up and dividing by $P_{t}$, i.e. the number of all perturbations annotated with this label
6. the result will be AP for the specific $t-p_{t}$ label-perturbation pair
7. repeat steps 2–6 for all perturbation profiles $P_{t}$ to obtain APs for all perturbations annotated with this label t
8. repeat steps 1–7 for all labels T to obtain APs for all label-perturbation pairs

The result will be a sparse P×T matrix of APs, where the element corresponding to a perturbation p and target t is equal to $AP_{t-p}$ if p is annotated with t and 0 otherwise. This matrix can be aggregated on a per-perturbation or per-label basis (for example, by taking the mean across rows or columns, correspondingly) depending on the downstream task. Per-label mAP will assess biological consistency of perturbations annotated with a specific label compared with perturbations annotated with other labels. Practically, this makes it possible to compare consistency of different label groupings for a given perturbation.

### Simulated data generation protocol

Simulations of the mAP performance were conducted by repeatedly generating control and treatment replicates by sampling features from a number of different normal distributions. Each treatment was simulated in 2,3 or 4 replicates, and 8, 16, or 32 replicates were simulated for each control. Between 100 and 5000 features were simulated. All features were simulated in the control by sampling from the standard normal distribution. Varying numbers of features were simulated in treatment replicates by sampling from a shifted normal distribution (μ = 1, σ = 1). Any remaining features in treatment replicates were sampled from the standard normal distribution. Each perturbation was considered correctly retrieved if its *p*-value was below 0.05.

### mp-value

The multidimensional perturbation value (mp-value)^14^ is a statistical metric designed to assess differences between treatments in various types of multidimensional screening data. It involves using principal component analysis (PCA) to transform the data, followed by calculating the Mahalanobis distance between treatment groups in this PCA-adjusted space. The significance of the mp-value is determined through permutation tests, a non-parametric approach that reshuffles replicate labels to assess the likelihood of observed differences occurring by chance.

### Cell Health dataset description and preprocessing

We used our published “Cell Health” dataset of Cell Painting^19^ images of CRISPR-Cas9 knockout perturbations of 59 genes, targeted by 119 guides in three different cell lines (A549, ES2, and HCC44)^32^. We used a subset of 100 guides that had exactly six replicates (two replicates in three different plates) in each cell line. We performed two types of profile normalization followed by feature selection using pycytominer^47^. The first preprocessing method included data standardization by subtracting means from feature values and dividing them by variance using the whole dataset. Alternatively, we used a robust version of standardization, which replaces mean and variance with median and median absolute deviation, correspondingly, and is applied on a per-plate basis (“Robust MAD”).

### cpg0004 dataset description and preprocessing

We used our previously published dataset “cpg0004-lincs” (abbreviated to cpg0004 here) that contains Cell Painting^19^ images of 1,327 small-molecule perturbations of A549 human cells^36^. The wells on each plate were perturbed with 56 different compounds in six different doses. Every compound was replicated 4 times per dose, with each replicated on a different plate. In this study, only the highest dose point of 10 μM was used. We normalized profiles per-plate by subtracting medians from feature values and dividing them by median absolute deviation (“Robust MAD”). We also applied feature selection using pycytominer^47^.

### cpg0016[orf] dataset description and preprocessing

We used the JUMP Consortium’s “cpg0016-jump[orf]” dataset^37^ (abbreviated to “cpg0016[orf]” here), which contains morphological profiles extracted from Cell Painting^19^ images of U2OS cells treated with 15,136 overexpression reagents (ORFs) encompassing 12,602 unique genes. We preprocessed profiles by first correcting for plate layout effects by subtracting means from feature values per well location and then regressing out cell counts from each feature with more than 100 unique values. After that, we normalized profiles per plate by subtracting medians from feature values and dividing them by median absolute deviation (“Robust MAD”). We also applied feature selection using pycytominer^47^ and corrected profiles for batch effects by a combination^48^ of the sphering transformation (computes a whitening transformation matrix based on negative controls and applies this transformation to the entire dataset) and Harmony^49^ (an iterative algorithm based on expectation-maximization that alternates between finding clusters with high diversity of batches, and computing mixture-based corrections within such clusters).

### Mitocheck data description and preprocessing

We accessed Mitocheck data from Image Data Resource (IDR)^50^ and processed images using a standard image analysis pipeline, which includes illumination correction (PyBasic^51^), segmentation (CellPose^52^), and single-cell feature extraction (CellProfiler^42^ and DeepProfiler^43^). We then processed single-cell image features using pycytominer^47^, normalizing the single-cells against a randomly sampled subset of negative control cells. Importantly, the MitoCheck consortium previously manually annotated about 2,916 cells to one of 16 phenotypic classes. We used this labeled dataset in all mAP score calculations. For complete data processing details, see ^53^ (https://github.com/WayScience/mitocheck_data).

### Perturb-seq dataset description and preprocessing

We used the public Perturb-seq^7–10^ mRNA profiling dataset of single cells treated with CRISPRi containing 10X single-cell gene expression reads, barcode identities, and activity readouts (Gene Expression Omnibus accession GSE132080)^54^. The experiment assessed how single-guide RNAs (sgRNAs) containing mismatches to their target sites attenuate expression levels of target genes^40^. Specifically, 25 genes involved in a diverse range of essential cell biological processes were targeted with 5–6 mismatched sgRNAs, covering the range from full to low activity, and 10 nontargeting controls. Each mismatched guide was characterized by its activity levels relative to the perfectly matched sgRNA targeting the same gene^40^. We performed single-cell profile normalization and feature selection using Seurat^55^.

## Supplementary Material

Supplement 1
