# Supplementary material for: A versatile information retrieval framework for evaluating profile strength and similarity
Source: bioRxiv. 2024 Dec 17:2024.04.01.587631. Originally published 2024 Apr 2. Preprint. [Version 2] doi: 10.1101/2024.04.01.587631 (PMC11014546; doi:10.1101/2024.04.01.587631)
Supplement: Supplementary file 1 [file media-1.pdf]

Supplemental Figures

A versatile information retrieval framework for evaluating profile strength and similarity

Alexandr A. Kalinin[1], John Arevalo[1], Erik Serrano[2], Loan Vulliard[3], Hillary Tsang[1], Michael Bornholdt[1], Suganya Sivagurunathan[1], Bartek Rajwa[4], Anne E. Carpenter[1], Gregory P. Way[2,*], Shantanu Singh[1,*]

[1]Imaging Platform, Broad Institute of MIT and Harvard, Cambridge MA, USA
[2]Department of Biomedical Informatics, University of Colorado School of Medicine, Aurora CO, USA
[3]Systems Immunology and Single-Cell Biology, German Cancer Research Center (DKFZ), Heidelberg, Germany
[4]Bindley Bioscience Center, Purdue University, West Lafayette IN, USA

* Corresponding Authors: gregory.way@cuanschutz.edu; shantanu@broadinstitute.org

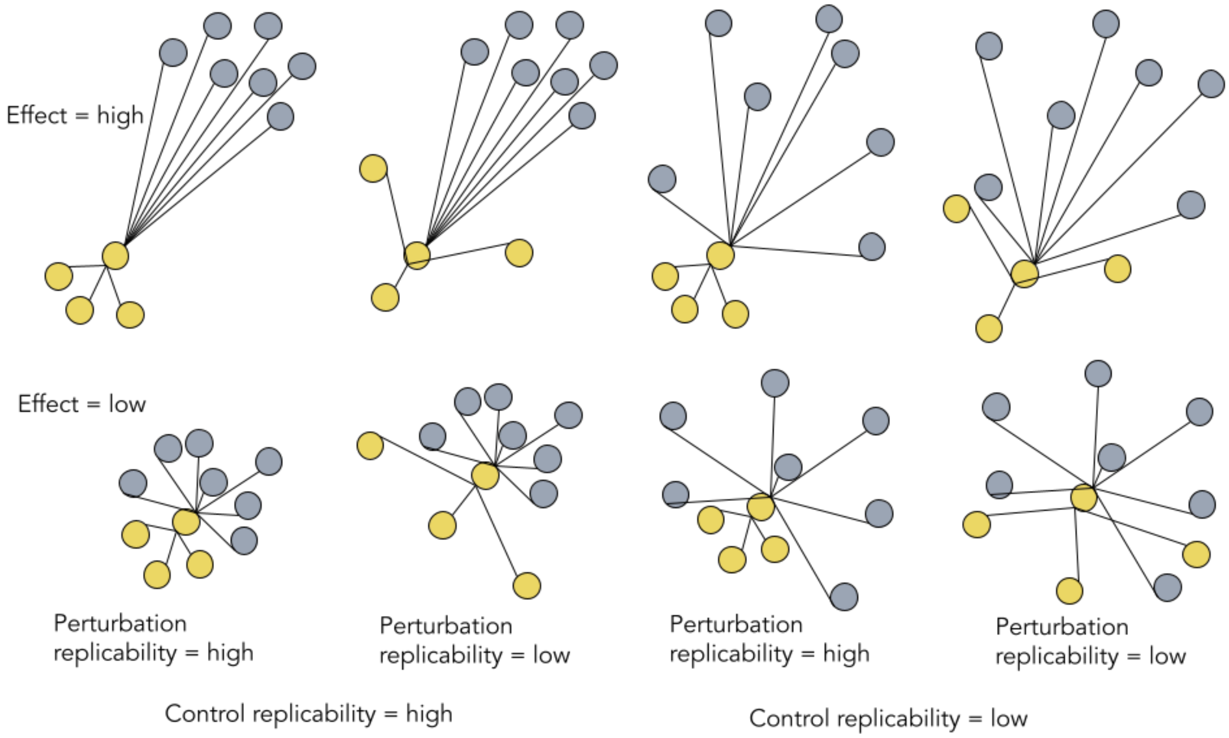

Figure S1. Schematic illustration of effect and replicability in phenotypic activity of a perturbation. Depending on effect strength induced by a perturbation and the amount of technical replicability variation, it may be nearly impossible to distinguish between effect and replicability.

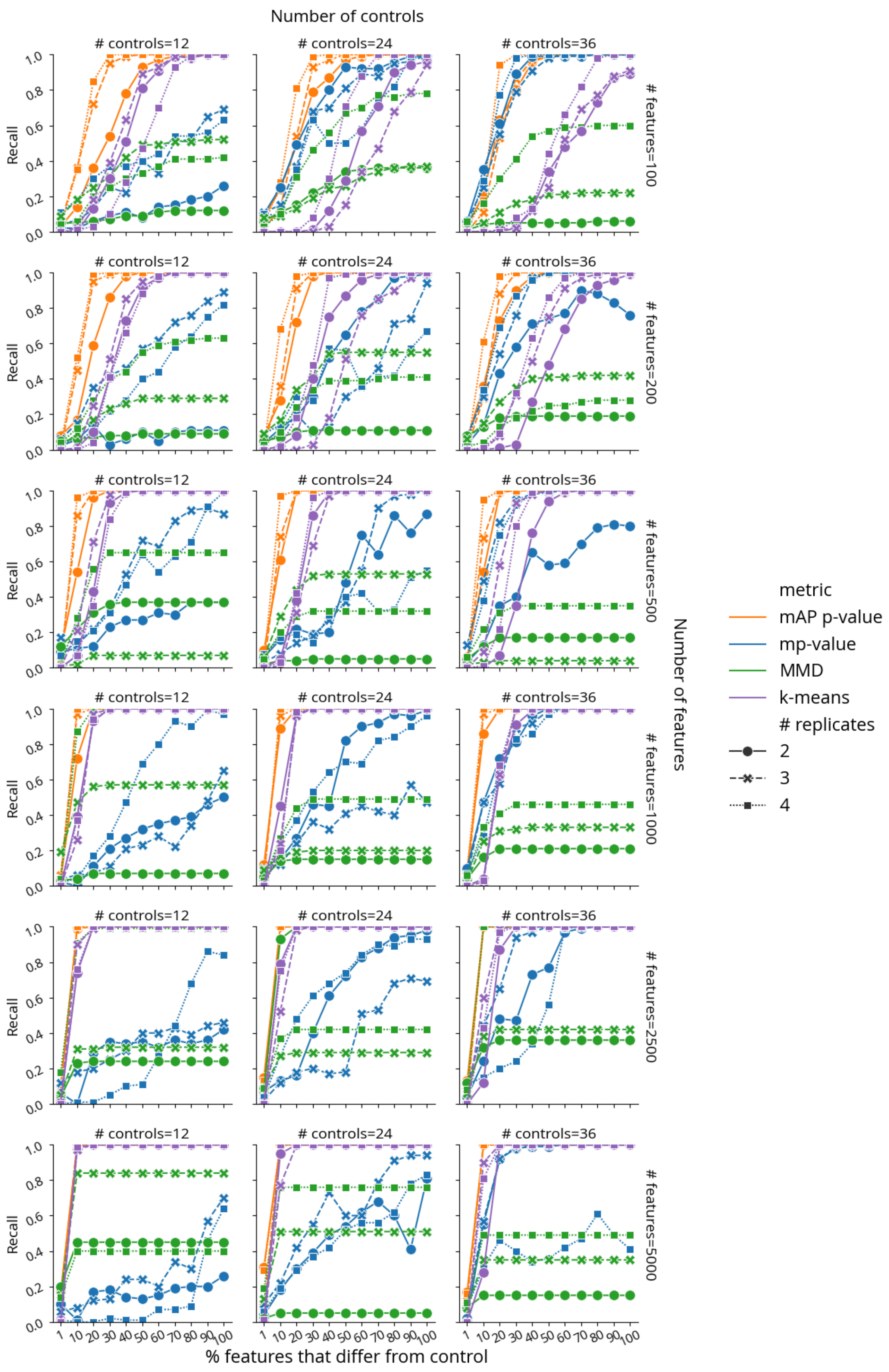

Number of controls
controls=12
controls=24
controls=36
Recall
features=100
features=200
features=500
features=1000
features=2500
features=5000
Number of features
% features that differ from control
metric
mAP p-value
mp-value
MMD
k-means
replicates
2
3
4

Figure S2. Benchmarking retrieval performance of mAP p-value (orange), mp-value (blue), MMD p-value (green), and k-means clustering (purple) for assessing phenotypic activity on simulated data, where unperturbed and perturbed features are sampled from $\mathcal{N}(0,1)$ and $\mathcal{N}(1,1)$, correspondingly. Recall indicates the percentage of 100 simulated perturbations under each condition that were called accurately by each method (as distinguishable from negative controls, or not). The horizontal axis probes what proportion of the features in the profile was different from controls. Marker and line styles indicate different numbers of replicates per perturbation (# replicates of 2, 3, and 4). Columns correspond to the different number of controls (# controls of 12, 24, and 36). Rows correspond to different profile sizes (# features being 100, 200, 500, 1000, 2500, and 5000).

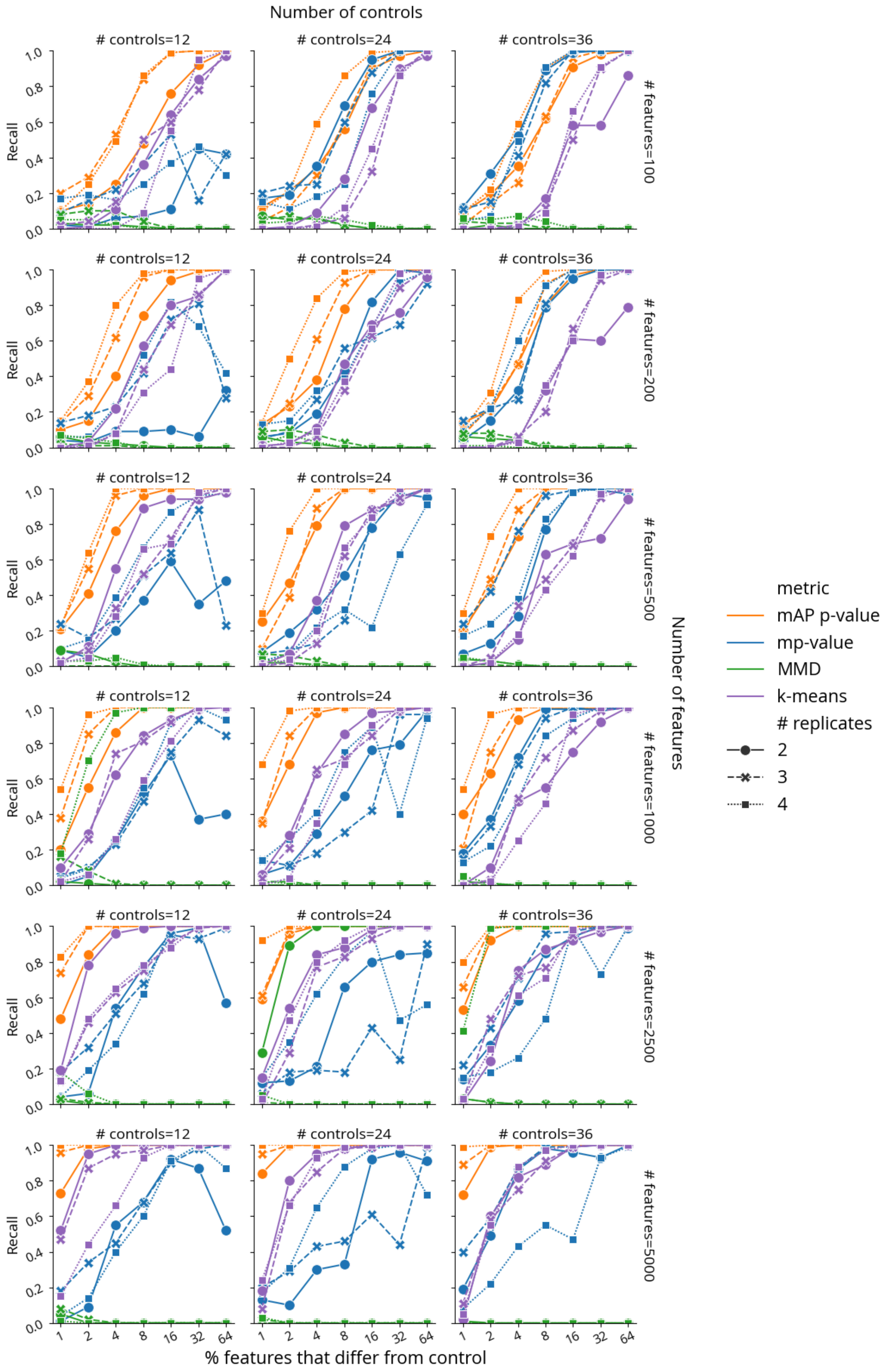

Number of controls
controls=12
controls=24
controls=36
Recall
features=100
features=200
features=500
features=1000
features=2500
features=5000
Number of features
% features that differ from control
metric
mAP p-value
mp-value
MMD
k-means
replicates
2
3
4

Figure S3. Benchmarking retrieval performance of mAP p-value (orange), mp-value (blue), and MMD p-value for phenotypic activity on simulated data, where unperturbed and perturbed features are sampled from $\mathcal{N}(0,1)$ and $\mathcal{N}(2,2)$, correspondingly. Recall indicates the percentage of 100 simulated perturbations under each condition that were called accurately by each method (as distinguishable from negative controls, or not). The horizontal axis probes what proportion of the features in the profile was different from controls. Marker and line styles indicate different numbers of replicates per perturbation (# replicates of 2, 3, and 4). Columns correspond to the different number of controls (# controls of 12, 24, and 36). Rows correspond to different profile sizes (# features being 100, 200, 500, 1000, 2500, and 5000).

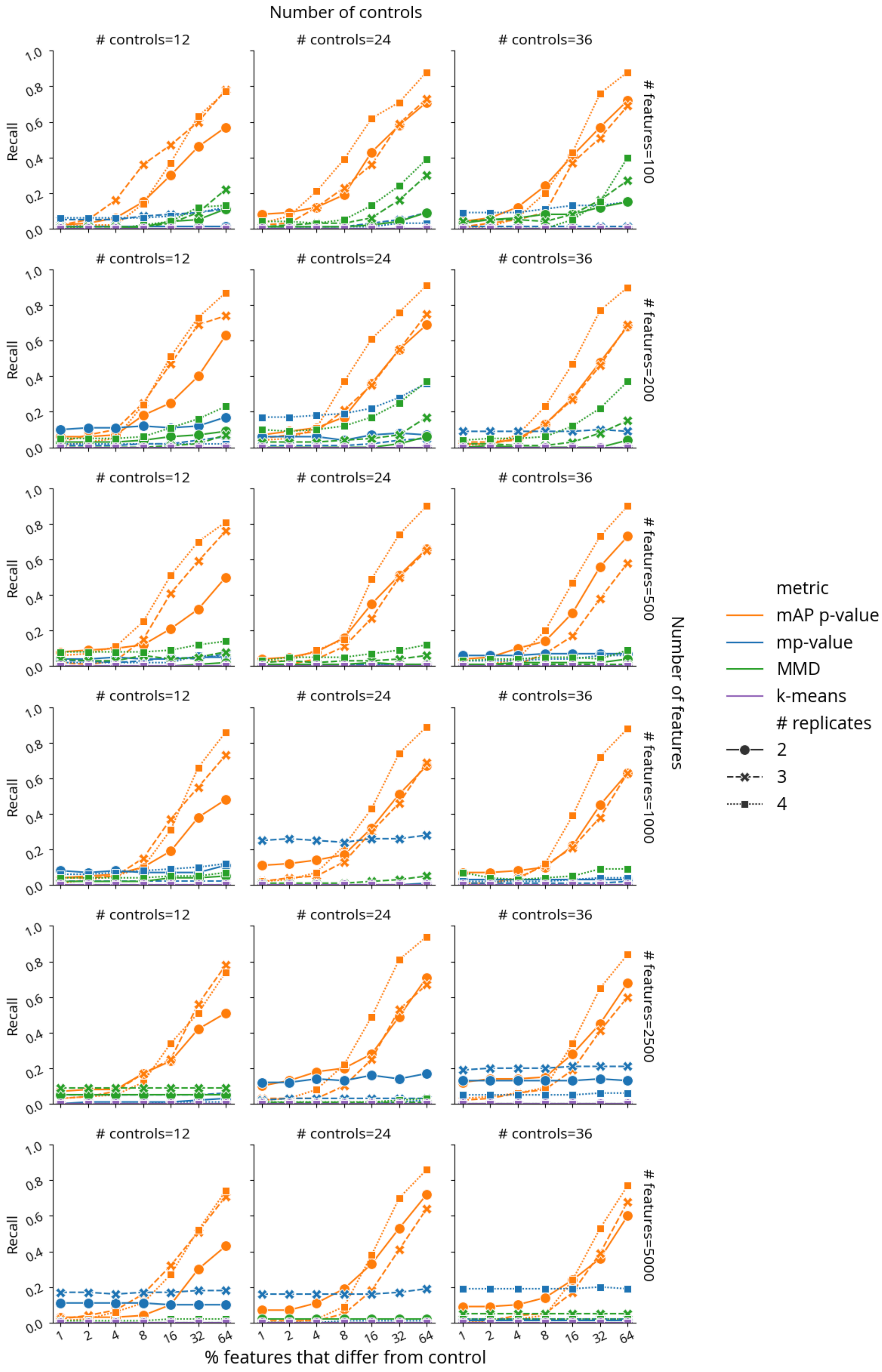
Number of controls
controls=12
controls=24
controls=36
Recall
features=100
features=200
features=500
features=1000
features=2500
features=5000
Number of features
metric
mAP p-value
mp-value
MMD
k-means
replicates
2
3
4
% features that differ from control

Figure S4. Benchmarking retrieval performance of mAP p-value (orange), mp-value (blue), and MMD p-value for phenotypic activity on simulated data, where unperturbed and perturbed features are sampled from Cauchy(0,1) and Cauchy(10,1), correspondingly. Recall indicates the percentage of 100 simulated perturbations under each condition that were called accurately by each method (as distinguishable from negative controls, or not). The horizontal axis probes what proportion of the features in the profile was different from controls. Marker and line styles indicate different numbers of replicates per perturbation (# replicates of 2, 3, and 4). Columns correspond to the different number of controls (# controls of 12, 24, and 36). Rows correspond to different profile sizes (# features being 100, 200, 500, 1000, 2500, and 5000).

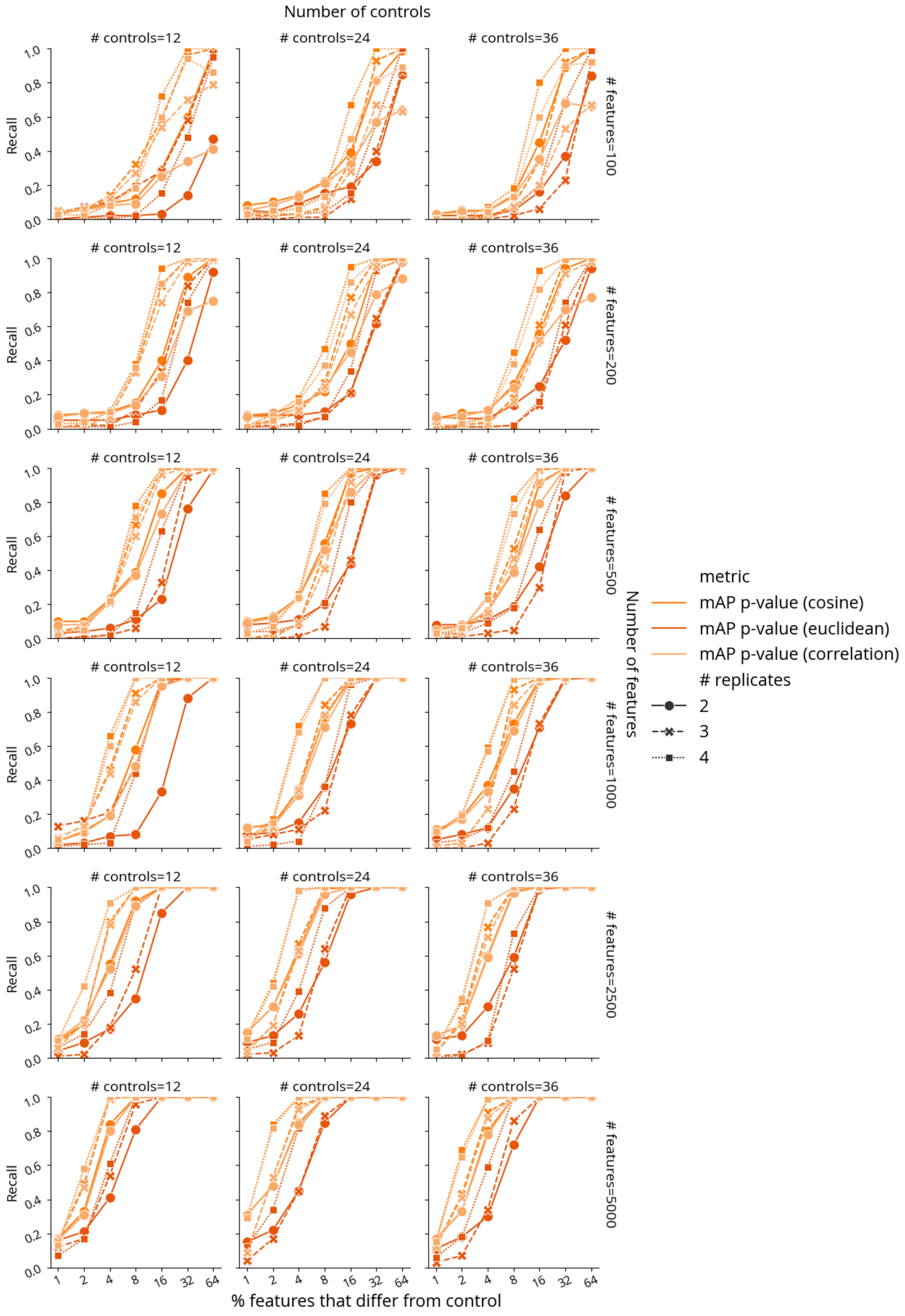
Number of controls
controls=12
controls=24
controls=36
Recall
features=100
features=200
features=500
features=1000
features=2500
features=5000
Number of features
% features that differ from control
metric
mAP p-value (cosine)
mAP p-value (euclidean)
mAP p-value (correlation)
replicates
2
3
4

Figure S5. Benchmarking retrieval performance of mAP p-value calculated using different distance metrics. We compared mAP p-value retrieval with the default choice of cosine distance (orange) against mAP p-value with Euclidean distance (dark orange) and correlation (light orange) for phenotypic activity on simulated data, where unperturbed and perturbed features are sampled from $\mathcal{N}(0,1)$ and $\mathcal{N}(1,1)$, correspondingly. Recall indicates the percentage of 100 simulated perturbations under each condition that were called accurately by each method (as distinguishable from negative controls, or not). The horizontal axis probes what proportion of the features in the profile was different from controls. Marker and line styles indicate different numbers of replicates per perturbation (# replicates of 2, 3, and 4). Columns correspond to the different number of controls (# controls of 12, 24, and 36). Rows correspond to different profile sizes (# features being 100, 200, 500, 1000, 2500, and 5000).

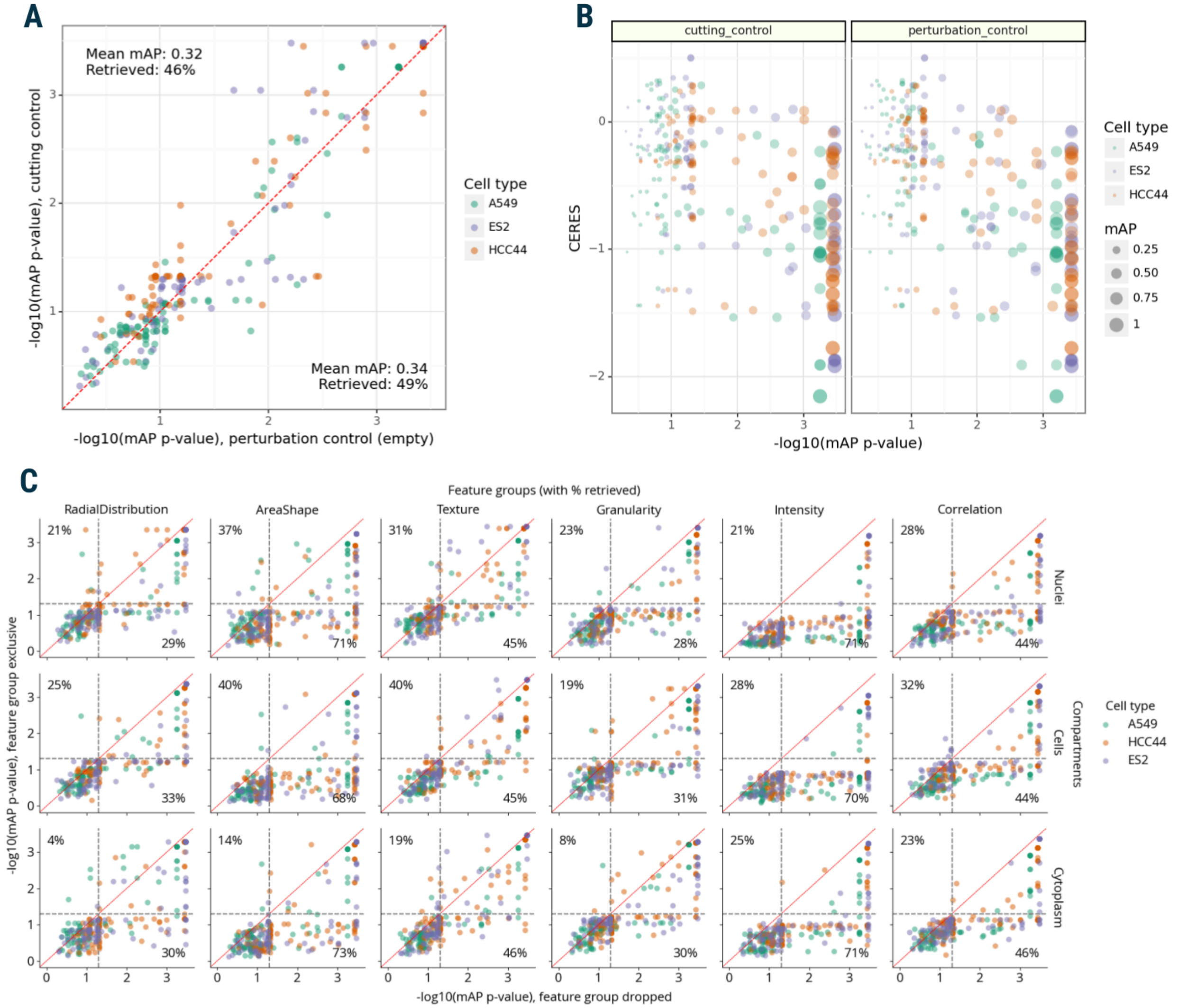

Figure S6. Additional results for mAP application to morphological profiling using Cell Health data. **A**: Correlation between mAP *p*-values that assessed the phenotypic activity of perturbations by guide replicate retrievability against non-targeting cutting controls and perturbation controls (empty wells). **B**: Correlation between mAP *p*-values that assessed the phenotypic activity of perturbations by guide replicate retrievability against non-targeting cutting controls and perturbation controls (empty wells). **C**: mAP *p*-values calculated to assess influence of individual fluorescent channels on guide phenotypic activity against controls by either dropping a channel or including only that single channel (percent retrieved is shown for each axis), these results can be compared to 49% retrieved when all channels' data is available (on average across all three cell lines, as in **B**). **D**: mAP is calculated to assess the phenotypic consistency of guides annotated with related target genes (against guides annotated with other genes) in three cell lines individually.

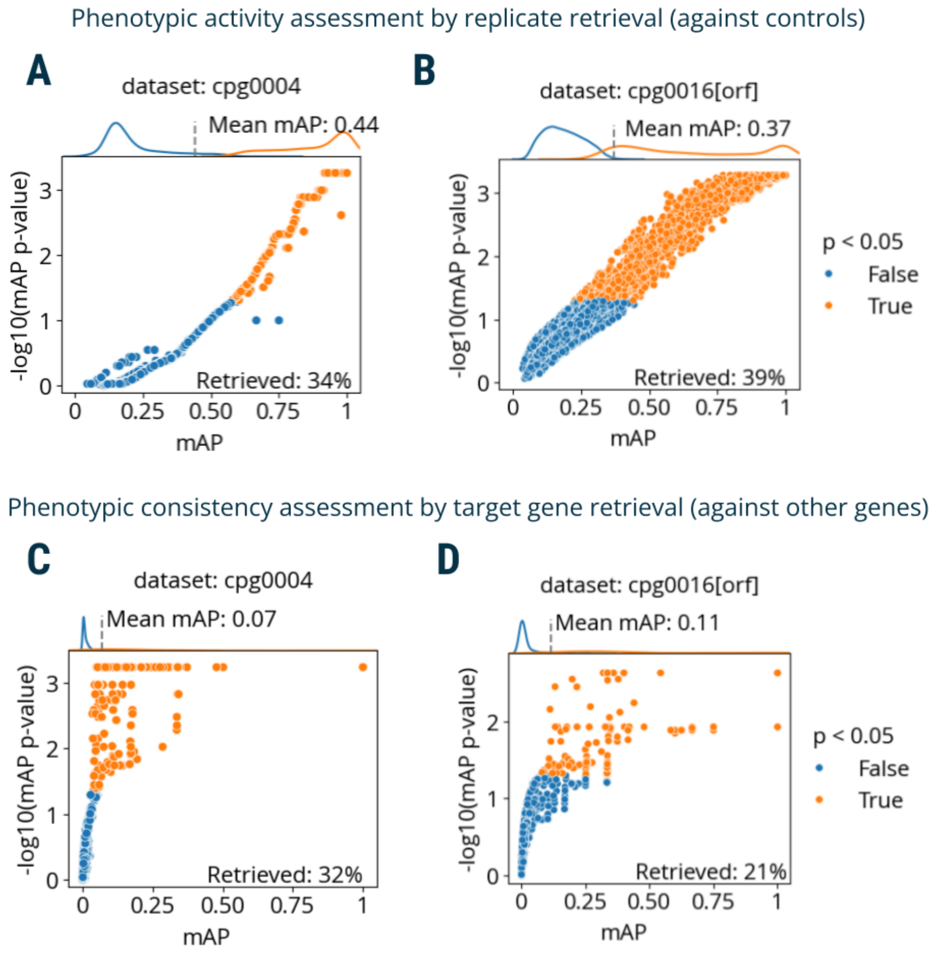

Figure S7. Additional results for mAP application to morphological profiling of small molecule (cpg0004) and gene overexpression (cpg0016[orf]) perturbation datasets. **A**: mAP is calculated to assess the phenotypic activity of compounds by replicate retrievability against controls in cpg0004 data. **B**: mAP is calculated to assess the phenotypic activity of gene overexpression reagents by replicate retrievability against controls in cpg0016[orf] data. **C**: mAP is calculated to assess the phenotypic consistency of compounds annotated with related target genes (against compounds annotated with other genes) in cpg0004 data. **D**: mAP is calculated to assess the phenotypic consistency of compounds annotated with related target genes (against compounds annotated with other genes) in cpg0004 data.

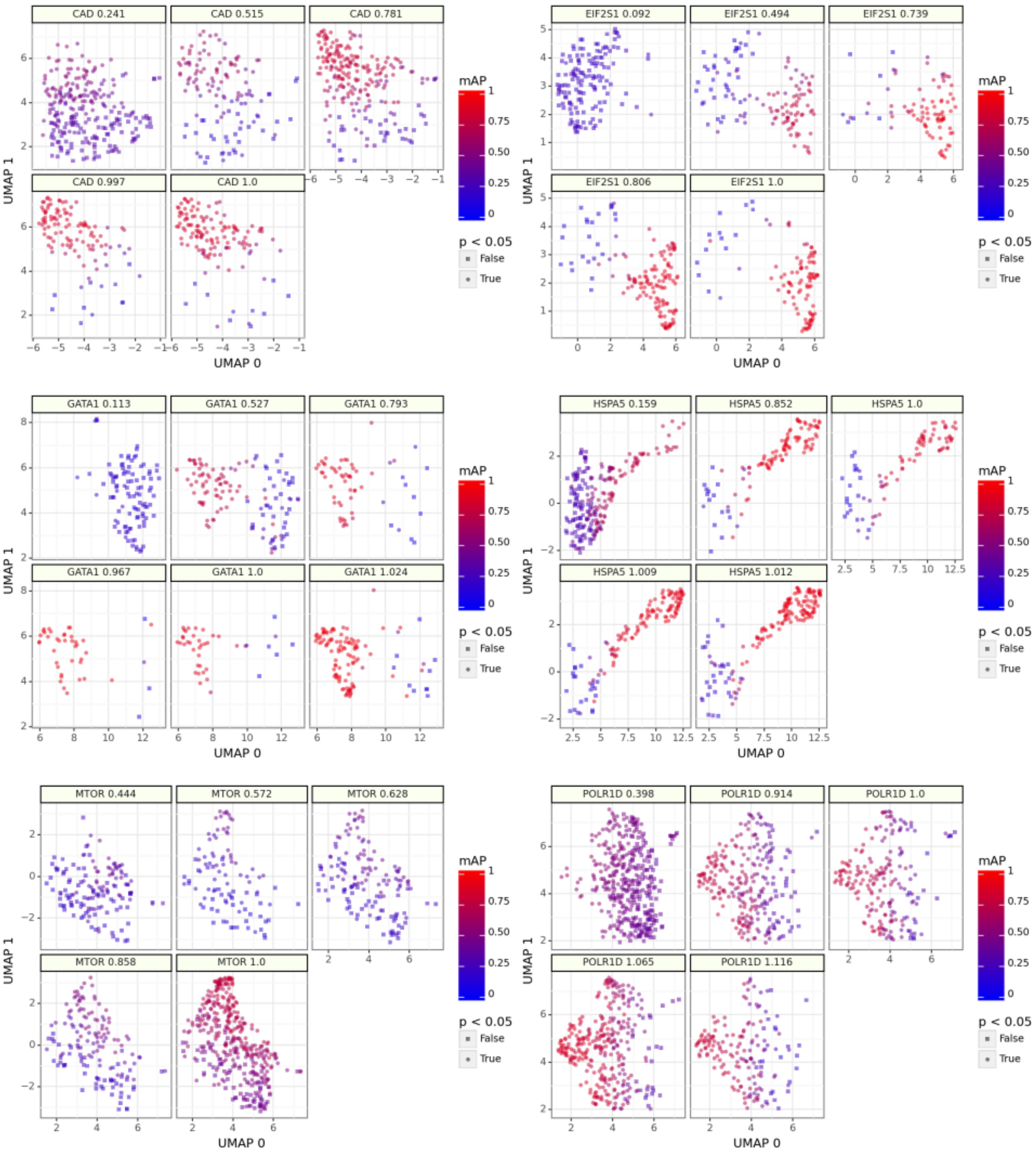

Figure S8. Single-cell profile AP scores assessing phenotypic activity for individual guides with varying mismatches relative to the perfect-matching guide (a subset of data shown in Figure 5A). Guides with higher relative activity scores demonstrate higher single-cell AP values. UMAP embeddings were created using n_neighbors=30 and cosine similarity as a distance measure.

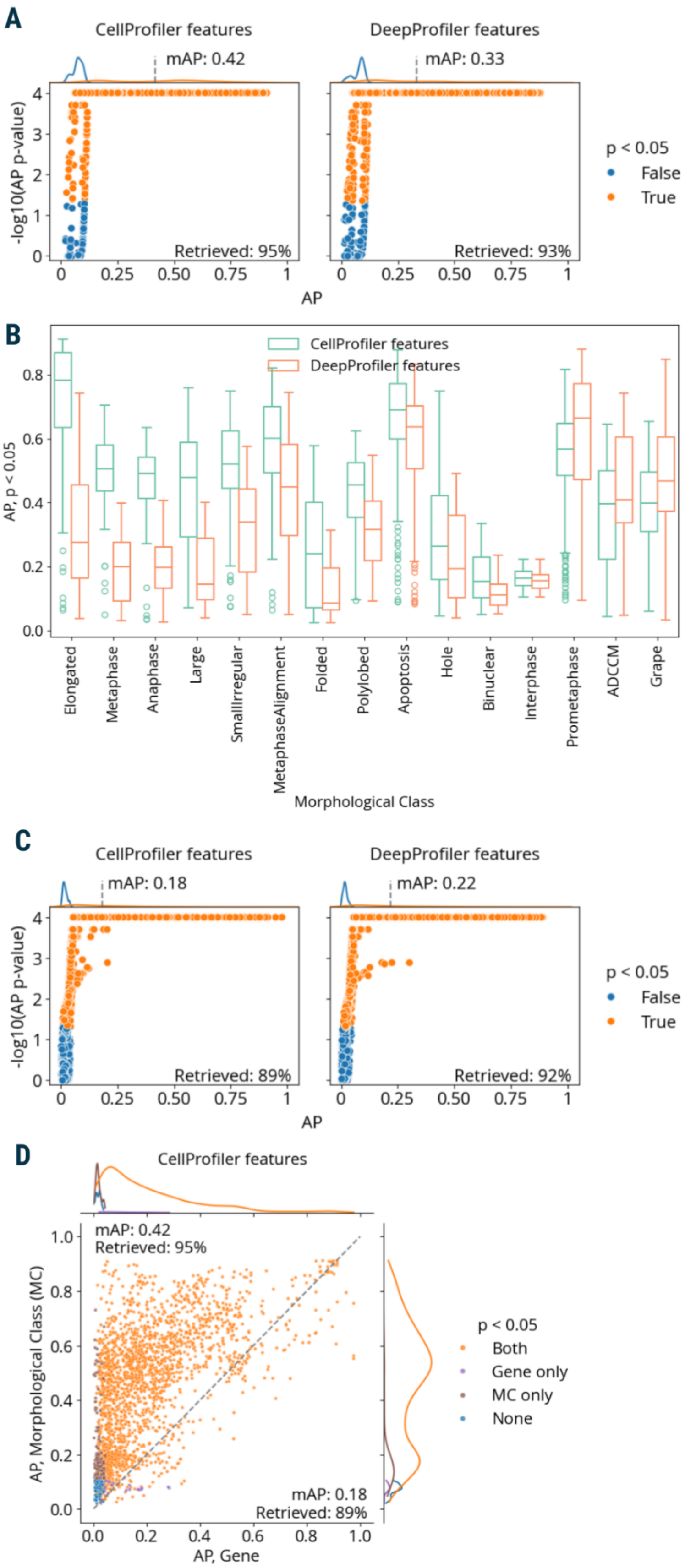
A
CellProfiler features
DeepProfiler features
mAP: 0.42
mAP: 0.33
-log10(AP p-value)
Retrieved: 95%
Retrieved: 93%
AP
p < 0.05
False
True
B
CellProfiler features
DeepProfiler features
AP, p < 0.05
Elongated
Metaphase
Anaphase
Large
SmallIrregular
MetaphaseAlignment
Folded
Polylobed
Apoptosis
Hole
Binuclear
Interphase
Prometaphase
ADCCM
Grape
Morphological Class
C
CellProfiler features
DeepProfiler features
mAP: 0.18
mAP: 0.22
-log10(AP p-value)
Retrieved: 89%
Retrieved: 92%
AP
p < 0.05
False
True
D
CellProfiler features
mAP: 0.42
Retrieved: 95%
mAP: 0.18
Retrieved: 89%
AP, Morphological Class (MC)
AP, Gene
p < 0.05
Both
Gene only
MC only
None

Figure S9. Additional results for mAP application to single-cell profiling using Mitocheck morphological data. **A**: AP scores can be used to evaluate the power of CellProfiler and DeepProfiler features to classify morphological classes in Mitocheck data. AP scores capture the ability to retrieve single cells annotated with the same morphological class against negative controls. **B**: A subset of AP scores with $p < 0.05$ from panel A is shown to evaluate the power of CellProfiler and DeepProfiler features to classify specific morphological classes. **C**: AP scores can be used to evaluate the power of CellProfiler and DeepProfiler features to classify target genes in Mitocheck data. AP scores capture the ability to retrieve single cells annotated with the same target gene against negative controls. **D**: A subset of AP scores obtained using CellProfiler features from panels **A** and **C** is shown to compare retrieval of morphological classes and target genes.

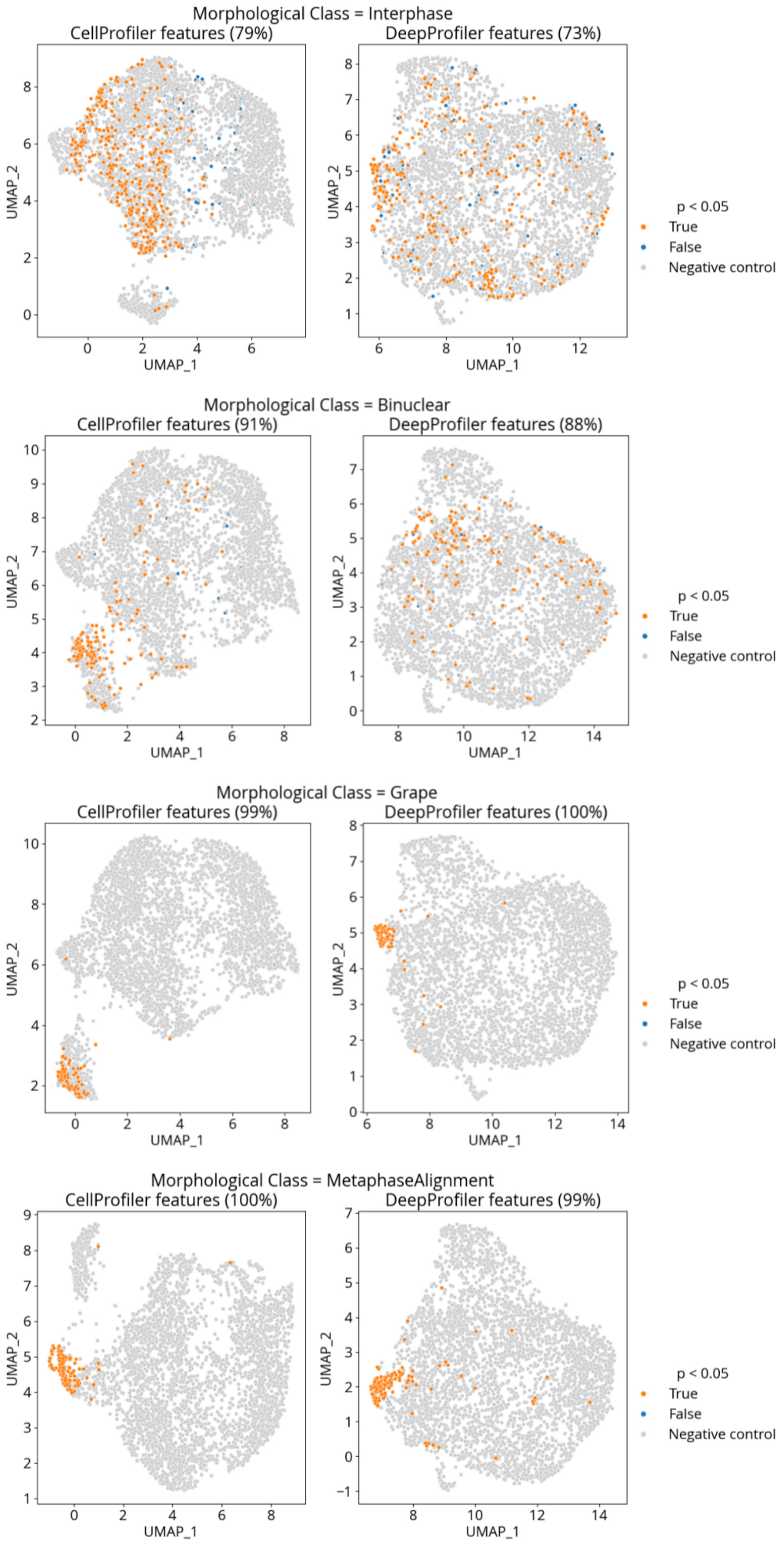

Morphological Class = Interphase
CellProfiler features (79%)
DeepProfiler features (73%)
Morphological Class = Binuclear
CellProfiler features (91%)
DeepProfiler features (88%)
Morphological Class = Grape
CellProfiler features (99%)
DeepProfiler features (100%)
Morphological Class = MetaphaseAlignment
CellProfiler features (100%)
DeepProfiler features (99%)
UMAP_1
UMAP_2
p < 0.05
True
False
Negative control

Figure S10. Single-cell profile AP scores assessing phenotypic activity for exemplar morphological classes in Mitocheck data with CellProfiler and DeepProfiler features (a subset of data shown in Figure 5C). Classes with higher retrieval rates (Grape, MetaphaseAlignment) demonstrate tighter clustering compared to those with lower rates (Interphase, Binuclear with DeepProfiler features). UMAP embeddings were created using n_neighbors=30 and cosine similarity as a distance measure.
