# Supplementary material for: A versatile information retrieval framework for evaluating profile strength and similarity
Source: bioRxiv. 2024 Apr 2:2024.04.01.587631. Preprint. [Version 1] doi: 10.1101/2024.04.01.587631 (PMC11014546; doi:10.1101/2024.04.01.587631)
Supplement: Supplementary file 1 [file media-1.pdf]

Supplemental Figures

A versatile information retrieval framework for evaluating profile strength and similarity

Alexandr A. Kalinin[1], John Arevalo[1], Loan Vulliard[2], Erik Serrano[3], Hillary Tsang[1], Michael Bornholdt[1], Bartek Rajwa[4], Anne E. Carpenter[1], Gregory P. Way[3,*], Shantanu Singh[1,*]

[1]Imaging Platform, Broad Institute of MIT and Harvard, Cambridge MA, USA
[2]Systems Immunology and Single-Cell Biology, German Cancer Research Center (DKFZ), Heidelberg, Germany
[3]Department of Biomedical Informatics, University of Colorado School of Medicine, Aurora CO, USA
[4]Bindley Bioscience Center, Purdue University, West Lafayette IN, USA

* Corresponding Authors: gregory.way@cuanschutz.edu; shantanu@broadinstitute.org

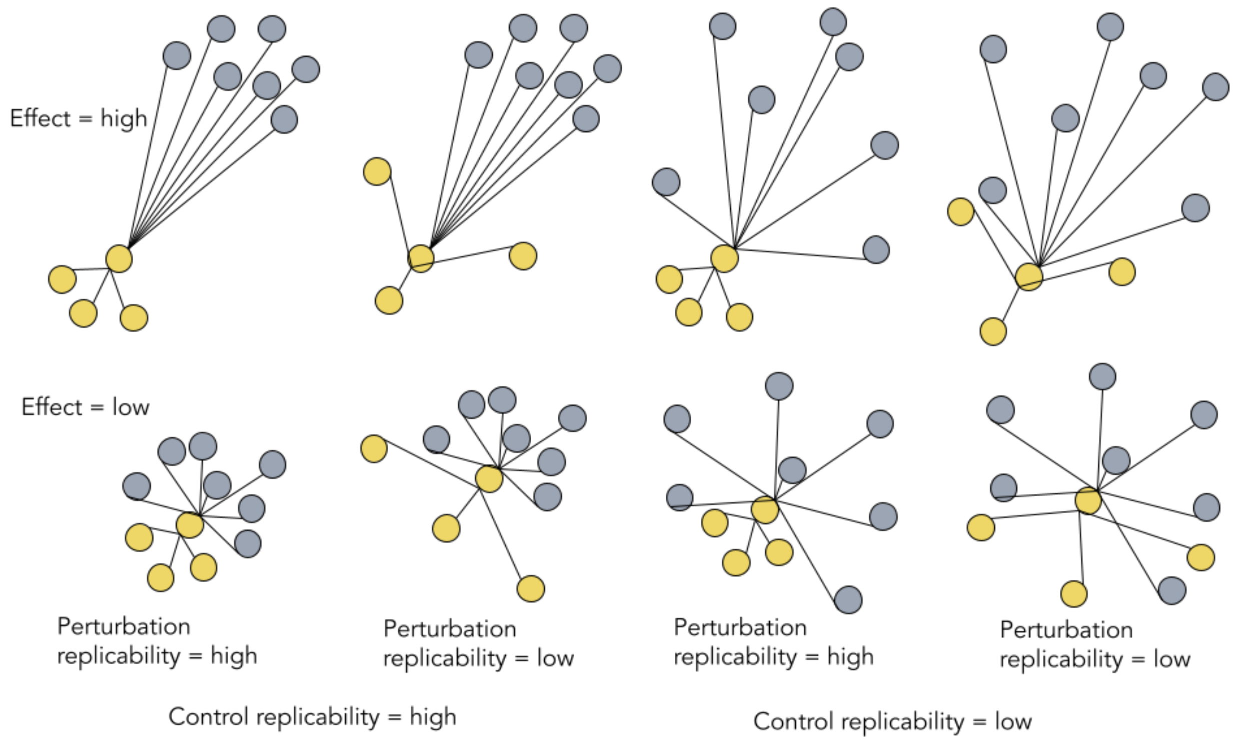

Figure S1. Schematic illustration of effect and replicability in phenotypic activity of a perturbation. Depending on effect strength induced by a perturbation and the amount of technical replicability variation, it may be nearly impossible to distinguish between effect and replicability.

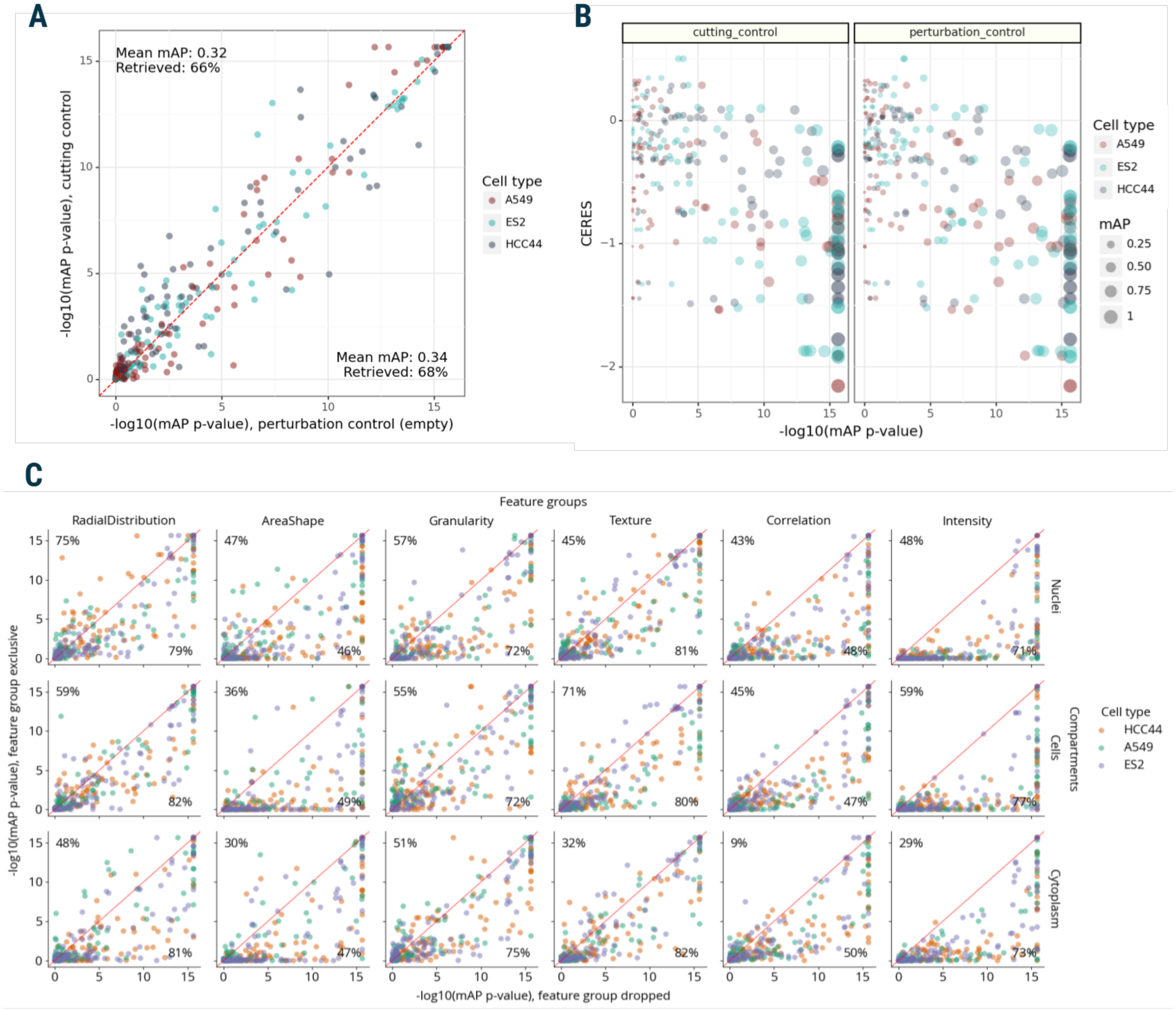

Figure S2. Additional results for mAP application to morphological profiling using Cell Health data. **A**: Correlation between mAP *p*-values that assessed the phenotypic activity of perturbations by guide replicate retrievability against non-targeting cutting controls and perturbation controls (empty wells). **B**: Correlation between mAP *p*-values that assessed the phenotypic activity of perturbations by guide replicate retrievability against non-targeting cutting controls and perturbation controls (empty wells). **C**: mAP *p*-values calculated to assess influence of individual fluorescent channels on guide phenotypic activity against controls by either dropping a channel or including only that single channel (percent retrieved is shown for each axis), these results can be compared to 68% retrieved when all channels' data is available (on average across all three cell lines, as in **B**). **D**: mAP is calculated to assess the phenotypic consistency of guides annotated with related target genes (against guides annotated with other genes) in three cell lines individually.

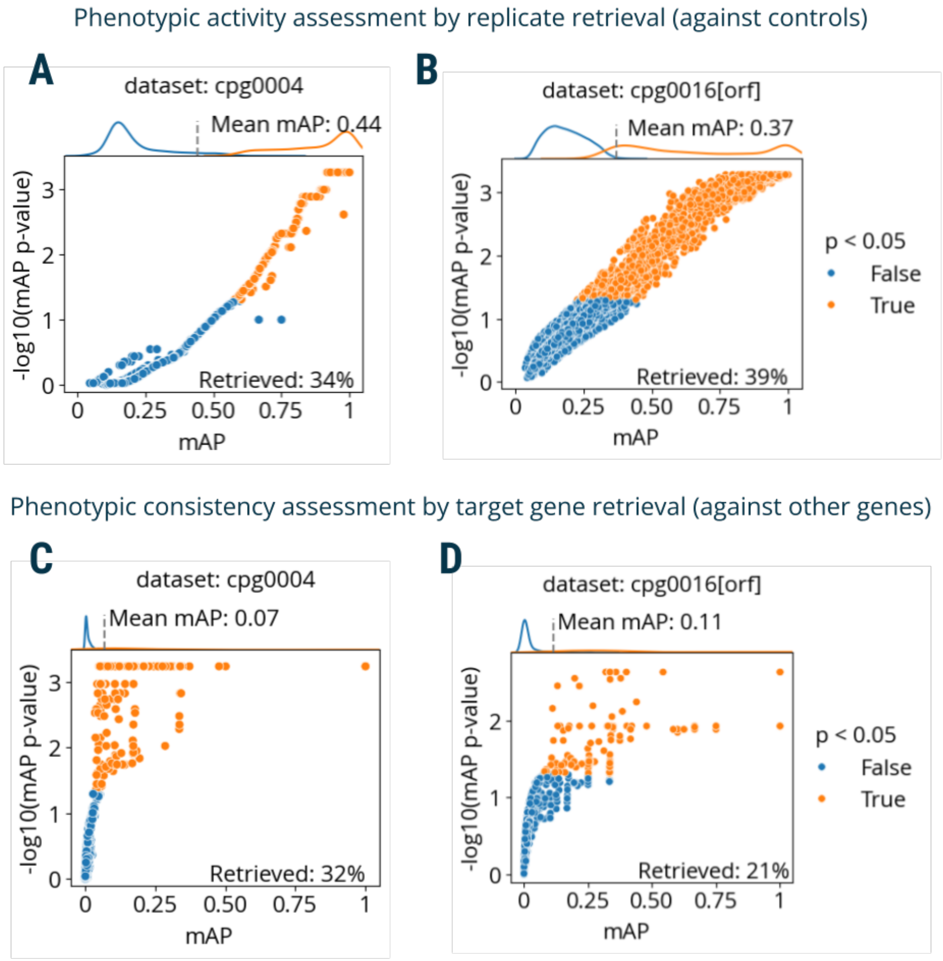

Figure S3. Additional results for mAP application to morphological profiling of small molecule (cpg0004) and gene overexpression (cpg0016[orf]) perturbation datasets. **A**: mAP is calculated to assess the phenotypic activity of compounds by replicate retrievability against controls in cpg0004 data. **B**: mAP is calculated to assess the phenotypic activity of gene overexpression reagents by replicate retrievability against controls in cpg0016[orf] data. **C**: mAP is calculated to assess the phenotypic consistency of compounds annotated with related target genes (against compounds annotated with other genes) in cpg0004 data. **D**: mAP is calculated to assess the phenotypic consistency of compounds annotated with related target genes (against compounds annotated with other genes) in cpg0004 data.

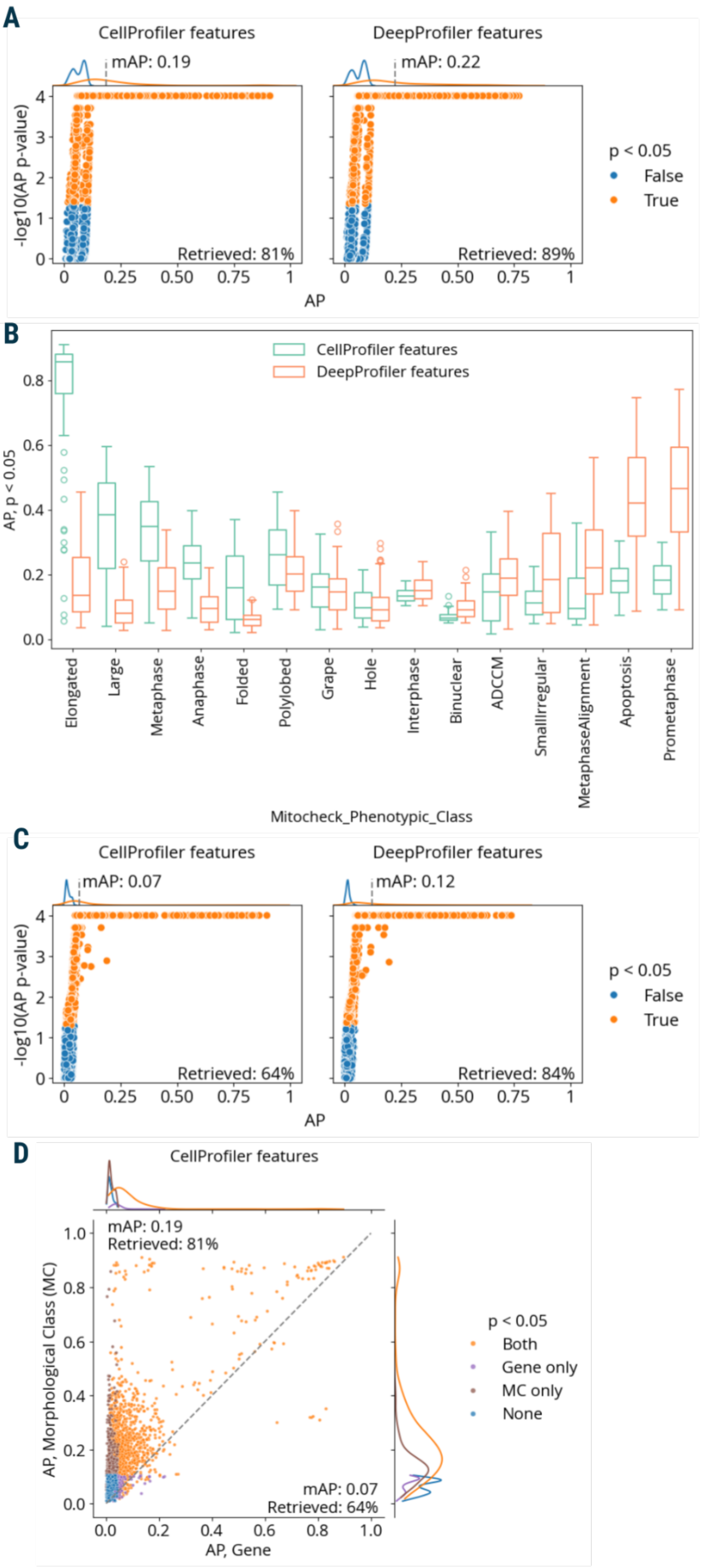
A
CellProfiler features
DeepProfiler features
mAP: 0.19
mAP: 0.22
-log10(AP p-value)
Retrieved: 81%
Retrieved: 89%
AP
p < 0.05
False
True
B
CellProfiler features
DeepProfiler features
AP, p < 0.05
Elongated
Large
Metaphase
Anaphase
Folded
Polylobed
Grape
Hole
Interphase
Binuclear
ADCCM
SmallIrregular
MetaphaseAlignment
Apoptosis
Prometaphase
Mitocheck_Phenotypic_Class
C
CellProfiler features
DeepProfiler features
mAP: 0.07
mAP: 0.12
-log10(AP p-value)
Retrieved: 64%
Retrieved: 84%
AP
p < 0.05
False
True
D
CellProfiler features
mAP: 0.19
Retrieved: 81%
AP, Morphological Class (MC)
mAP: 0.07
Retrieved: 64%
AP, Gene
p < 0.05
Both
Gene only
MC only
None

Figure S4. Additional results for mAP application to single-cell profiling using Mitocheck morphological data. **A**: AP scores are calculated can be used to evaluate the power of CellProfiler and DeepProfiler features to classify morphological classes in Mitocheck data. AP scores capture the ability to retrieve single cells annotated with the same morphological class against negative controls. **B**: A subset of AP scores with $p < 0.05$ from panel A is shown to evaluate the power of CellProfiler and DeepProfiler features to classifying specific morphological classes. **C**: AP scores are calculated can be used to evaluate the power of CellProfiler and DeepProfiler features to classify target genes in Mitocheck data. AP scores capture the ability to retrieve single cells annotated with the same target gene against negative controls. **D**: A subset of AP scores obtained using CellProfiler features from panels **A** and **C** is shown to compare retrieval of morphological classes and target genes.
